# Differential Effects of Rapamycin and Dexamethasone in Mouse Models of Established Allergic Asthma

**DOI:** 10.1371/journal.pone.0054426

**Published:** 2013-01-17

**Authors:** Elizabeth M. Mushaben, Eric B. Brandt, Gurjit K. Khurana Hershey, Timothy D. Le Cras

**Affiliations:** 1 Division of Pulmonary Biology, Department of Pediatrics, Cincinnati Children’s Hospital, University of Cincinnati School of Medicine, Cincinnati, Ohio, United States of America; 2 Division of Asthma Research, Department of Pediatrics, Cincinnati Children’s Hospital, University of Cincinnati School of Medicine, Cincinnati, Ohio, United States of America; Leiden University Medical Center, The Netherlands

## Abstract

The mammalian target of rapamycin (mTOR) plays an important role in cell growth/differentiation, integrating environmental cues, and regulating immune responses. Our lab previously demonstrated that inhibition of mTOR with rapamycin prevented house dust mite (HDM)-induced allergic asthma in mice. Here, we utilized two treatment protocols to investigate whether rapamycin, compared to the steroid, dexamethasone, could inhibit allergic responses during the later stages of the disease process, namely allergen re-exposure and/or during progression of chronic allergic disease. In protocol 1, BALB/c mice were sensitized to HDM (three i.p. injections) and administered two intranasal HDM exposures. After 6 weeks of rest/recovery, mice were re-exposed to HDM while being treated with rapamycin or dexamethasone. In protocol 2, mice were exposed to HDM for 3 or 6 weeks and treated with rapamycin or dexamethasone during weeks 4–6. Characteristic features of allergic asthma, including IgE, goblet cells, airway hyperreactivity (AHR), inflammatory cells, cytokines/chemokines, and T cell responses were assessed. In protocol 1, both rapamycin and dexamethasone suppressed goblet cells and total CD4^+^ T cells including activated, effector, and regulatory T cells in the lung tissue, with no effect on AHR or total inflammatory cell numbers in the bronchoalveolar lavage fluid. Rapamycin also suppressed IgE, although IL-4 and eotaxin 1 levels were augmented. In protocol 2, both drugs suppressed total CD4^+^ T cells, including activated, effector, and regulatory T cells and IgE levels. IL-4, eotaxin, and inflammatory cell numbers were increased after rapamycin and no effect on AHR was observed. Dexamethasone suppressed inflammatory cell numbers, especially eosinophils, but had limited effects on AHR. We conclude that while mTOR signaling is critical during the early phases of allergic asthma, its role is much more limited once disease is established.

## Introduction

Allergic asthma is a heterogeneous disease characterized by airway hyperreactivity (AHR), inflammation, goblet cell metaplasia, and increases in Th2 cytokines and IgE [1], [2], [3], [4]. Although current therapies such as glucocorticoids and bronchodilators are effective in suppressing symptoms in some patients, not all asthmatic patients respond to these therapies [1]. As the prevalence of asthma continues to rise, especially in children [1], [5], [6], it is imperative that the mechanisms underlying this disease be identified.

For some patients, allergic asthma is an ongoing disease, but for others, asthma symptoms only develop when patients are exposed to seasonal allergens or are exposed to a stimulus that provokes their asthma symptoms. Asthma exacerbations are a major problem and account for a high proportion of emergency room visits, hospitalizations and healthcare related cost [7], [8]. Prevention of these exacerbations or reversal of chronic, established allergic disease would help improve disease management and reduce both hospitalizations and deaths from acute asthma attacks.

Mammalian target of rapamycin (mTOR) signaling occurs downstream of the PI3K-signaling cascade and is known to play a major role in growth/differentiation, cell metabolism, and survival in many different cell types [9], [10]. More recent work has demonstrated an important role for mTOR in T cell proliferation and differentiation [11], [12], [13]. An inhibitor of mTOR, rapamycin, is already used clinically as an immunosuppressant to prevent organ rejection after transplantation [14], [15]. In addition, the use of rapamycin in patients suffering from the destructive lung disease, lymphangioleiomyomatosis [16], has demonstrated promise in its ability to reduce disease symptoms and stabilize lung function [17]. Previously, our lab demonstrated that inhibition of mTOR with rapamycin prevented allergic asthma in a mouse model induced by exposure to the allergen, house dust mite (HDM). In these studies, rapamycin prevented the allergic response and still suppressed many key asthma characteristics after allergic sensitization was established [18]. Although this study showed that mTOR inhibition could suppress allergic asthma early in the disease process, the role of mTOR during allergen re-exposure and chronic, established allergic disease remained unclear.

The goal of this study was to determine whether inhibition of mTOR with rapamycin would attenuate key characteristics of allergic asthma in two models that addressed chronic/established disease, namely allergen re-exposure and disease progression. In addition to rapamycin, mice were also treated with the steroid, dexamethasone, for comparison purposes since steroids are currently a mainstay therapy for chronic asthma [1]. We hypothesized that rapamycin and dexamethasone would suppress asthma exacerbations during allergen re-exposure and suppress progressive/ongoing allergic disease by inhibiting T cells. To test this hypothesis, mice in protocol one, which was designed to mimic the effects of allergen re-exposure in a previously sensitized individual, were sensitized to HDM by i.p. injection and then exposed to intranasal HDM to induce asthma. Then, after a 6 week rest/recovery period, mice were re-exposed to HDM while being treated with rapamycin or dexamethasone. In protocol two, to address the role of mTOR in chronic/established allergic asthma, mice were exposed to HDM for 6 weeks and treated with rapamycin or dexamethasone from weeks 4 to 6 of the exposure period. Endpoints assessed included allergen specific IgE, AHR, inflammatory cells, goblet cell metaplasia, cytokine/chemokine levels, and T cell numbers.

## Materials and Methods

### Ethics Statement and Animal Treatment Protocols

Animal procedures and protocols were approved by the Animal Care and Use Committee at the Cincinnati Children’s Hospital Research Foundation (Cincinnati, OH) (Protocol Number: 1D02011). All procedures were performed under anesthesia to minimize suffering. Female BALB/c mice were purchased at 6–8 weeks of age from Charles River Laboratories (Wilmington, MA). The two different treatment protocols that were used in this study are described below, and each study was performed twice.

### Protocol 1: Allergen Re-Exposure Model

**Figure 1:**
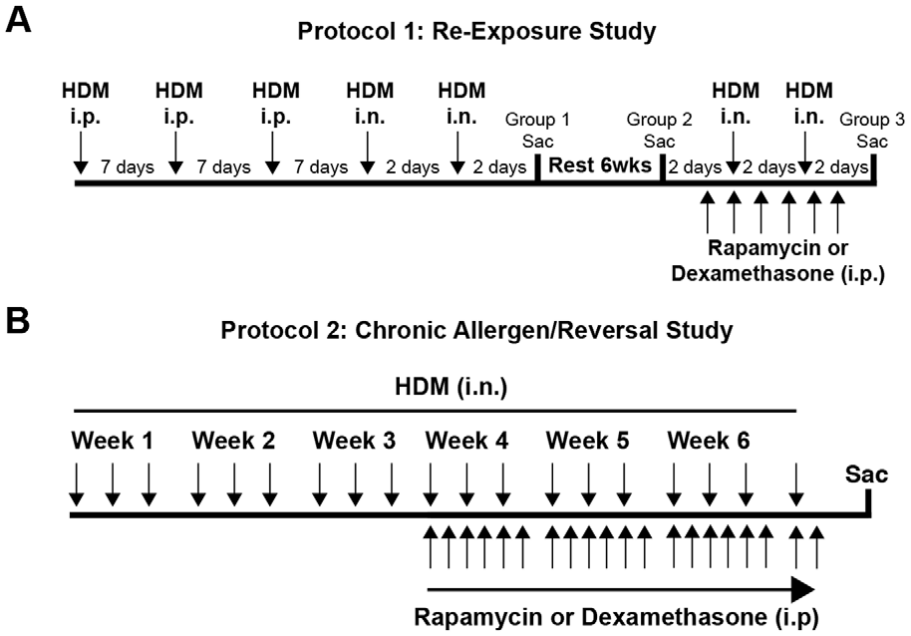
Study protocols. *A*: Protocol 1: Re-Exposure Study: Mice were sensitized to HDM by 3 i.p. injections followed by 2 intranasal HDM or saline (control) exposures. One group of mice (group 1) was sacrificed after the first round of allergen exposures while the other two groups rested/recovered for 6 weeks. After 6 weeks of rest, group 2 was sacrificed before allergen re-exposure. Mice in group 3 were re-exposed to intranasal HDM or saline (control) twice and were treated with rapamycin or dexamethasone during this exposure period. Mice were sacrificed 48 hours after the last HDM exposure. *B*: Protocol 2: Reversal Study: Mice were exposed to HDM intranasally 3 times per week for 3 or 6 weeks. Starting at week 4, groups of mice were treated with rapamycin or dexamethasone by i.p. injection six days per week for the remaining three weeks. Mice were sacrificed 48 hours after the last HDM exposure.

Mice were sensitized to HDM by three intraperitoneal (i.p.) injections (50 µg HDM in 100 µl saline; Greer Laboratories, Lenoir, NC) at 7 day intervals (Figure 1A). Two intranasal (i.n.) HDM exposures (50 µg HDM in 20 µl saline), 2 days apart, were administered 7 days after the last i.p. injection. One group of mice was sacrificed 48 hours after the last i.n. HDM exposure to determine the disease phenotype at this time point (Group 1). The remaining mice were allowed to rest from allergen exposure for 6 weeks, with no treatment. Mice from group 2 were sacrificed after 6 weeks of rest/recovery and before allergen re-exposure. Mice in group 3, which were previously exposed to HDM and rested for 6 weeks, were re-exposed to i.n. HDM (50 µg HDM in 20 µl saline) twice, 2 days apart. Rapamycin (4 mg/kg; LC Laboratories, Woburn, MA), dexamethasone (1 mg/kg; Sigma Aldrich, St. Louis, MO), or vehicle (0.25% PEG400, 0.25% Tween20 in dH_2_O) was administered by i.p. injection one day prior to i.n. HDM re-exposure and continued for 6 days for a total of 6 drug treatments (Figure 1A). All mice re-exposed to HDM (group 3) received vehicle ± rapamycin or dexamethasone.

### Protocol 2: Chronic Allergen/Reversal Model

Mice were exposed to intranasal doses of HDM (50 µg in 20 ul saline) or saline (0.9% NaCl, 20 µl; control group) 3 times a week for 3 or 6 weeks (Figure 1B). Another group of mice were treated with rapamycin (4 mg/kg) or dexamethasone (1 mg/kg) 6 days a week by i.p. injection starting at week 4 through week 6 of HDM exposure.

### Allergic Sensitization

To assess allergic sensitization, HDM-specific IgE and IgG1 levels were measured in the serum by ELISA as previously described [18], [19]. Briefly, an ELISA plate was coated overnight with 0.01% HDM in PBS. The next day, 200 µl of blocking solution (1% BSA in PBS) was added to the plate for 1 hour. Next, 50 µl of sample was added to the plate for 1 hour, followed by biotin-anti-mouse IgE or biotin-anti-mouse IgG1 (2.0 µg/ml, Pharmingen) for 1 hour. To detect biotin-labeled IgE or IgG1, streptavidin-HRP (1∶100, R & D Systems, Minneapolis, MN) was added to the plate for 30 minutes. A tetramethylbenzidine substrate reagent set (1∶1)(BD Biosciences, San Jose, CA) was added to detect levels of IgE or IgG1.

### Airway Hyperreactivity (AHR)

All mice were anesthetized with a mixture of ketamine/xylaxin/acepromazine (4∶1:1) by i.p. injection 48 hours after the last HDM exposure and before AHR was assessed. Airway resistance to increasing doses of methacholine was measured as previously described [18], [20]. Briefly, the trachea of each mouse was cannulated and connected to a flexiVent system (SCIREQ, Montreal, QC, Canada). 1×PBS (baseline) and increasing doses of methacholine (12.5, 25, and 50 mg/ml; acetyl-β-methylcholine chloride, Sigma, St. Louis, MO) were nebulized into the mouse lungs via the tracheotomy to measure airway resistance. The thoracic aorta was cut after lung mechanic measurements were finished so that blood could be collected for IgE and IgG1 measurements.

### Inflammatory Cells and Cytokine Levels

Inflammatory cells were collected and assessed in the bronchoalveolar lavage fluid (BALF) as previously described [18], [20]. Briefly, lungs were lavaged with 1 ml of 1×PBS containing BSA (1%) and EDTA (2 mM). The BALF was centrifuged (5,000 rpm), and the supernatant was collected and frozen at −80°C for cytokine measurements. Next, cells were resuspended in red blood cell lysis buffer (Sigma) to lyse any red blood cells. Cells were then centrifuged again, supernatant was removed, and cells were resuspended in 1×PBS+BSA (1%) and EDTA (2 mM). Total inflammatory BALF cells were counted using a hemacytometer and differential cell counts were determined by staining cytospin slides with Diff-Quick (Shandon Lipshaw, Pittsburgh, PA). Three hundred cells per slide were counted and then the percentages and total differential inflammatory cells numbers of macrophages, lymphocytes, neutrophils, and eosinophils were calculated. A multiplex biomarker panel (Cytokine/Chemokine Panel I) and Luminex xMAP technology (Millipore, Billerica, MA) were used to measure IL-4, IL-5, IL-13, and IL-17A cytokine levels as well as the chemokine, eotaxin 1 in the BALF. IFN-γ cytokine levels were measured in the BALF by ELISA according to the manufacturer’s instructions (Biolegend, San Diego, CA).

### Western Blot Analysis

Western blot analysis was performed on lung homogenates using the following antibodies: chloride channel, calcium activated, family member 3 (CLCA3, 1∶5,000; Abcam, Cambridge, MA) [18], [21], [22], Phosphorylated S6 (P-S6, 1∶1000; Cell Signaling, Danvers, MA), Phosphorylated Akt (P-Akt, 1∶1000; Cell Signaling, Danvers, MA), and SAM-pointed domain-containing Ets-like factor (SPDEF, 1∶5,000; in house). To control for protein loading, C4 Actin (1∶40,000; Seven Hills Bioreagents, Cincinnati, OH) levels were assessed and CLCA3, P-S6, and SPDEF levels were normalized to Actin levels. P-Akt was normalized to total Akt levels (1∶1000; Cell Signaling, Danvers, MA). HRP-conjugated secondary antibodies were goat anti-mouse and goat anti-rabbit (1∶10,000, Calbiochem). Chemiluminescence was detected using Luminata Forte Western HRP Substrate (Millipore, Billerica, MA). Data were imaged using an LAS4000 imaging system and quantitated with Multi Gauge 3 software (Fujifilm, Tokyo, Japan).

#### Muc5AC and α-SMA immunohistochemistry

Lungs were inflation fixed at a constant pressure (25 cmH_2_O) by tracheal installation of 4% paraformaldehyde, transferred to 70% ethanol after 24 hrs, and embedded in paraffin as previously described [18], [23], [24]. Immunostaining for Mucin 5AC (Muc5AC) and α-smooth muscle actin (α-SMA) were performed on 5 µm paraffin-embedded sections as previously described [18], [20], [23]. Briefly, for Muc5AC, slides were incubated with a primary Muc5AC mouse monoclonal antibody (diluted 1∶200, Thermo Scientific, Waltham, MA) overnight at 4°C, followed by a goat α mouse IgG1 secondary antibody (diluted 1∶200, Southern Biotech, Birmingham, AL). For α-SMA, slides were incubated with a primary α-SMA antibody (diluted 1∶20,000, Clone 1A4 Sigma, St. Louis,MO) overnight at 4°C, followed by a goat α mouse IgG2a secondary antibody (1∶200, Southern Biotech, Birmingham, AL). For both antibodies, signal was detected using the DAB method of detection. Digital images of Muc5AC and α-SMA immunostaining were obtained using a Zeiss Axioplan 2 microscope and camera (Carl Zeiss Microimaging, Thornwood, NY).

### Flow Cytometry

The upper right lung lobe was minced and incubated at 37°C for 25–30 minutes in 2 ml of RPMI 1640 containing Liberase DL (0.5 mg/ml; Roche Diagnostics, Idianapolis, IN) and DNAse I (0.5 mg/ml; Sigma, St Louis, MO). Lung cells were passed through a 70 µm cell strainer and the strainer washed with 5 ml of RPMI+DNAse I media. Cells were centrifuged and resuspended in 2 ml of RPMI before counting with a hemacytometer and viability confirmed by trypan blue exclusion. Approximately 500,000 lung cells were transferred to a 96 well plate with V shaped bottom on ice, centrifuged and resuspended in 1×PBS containing FcBlock (2.4G2 mAb). Lung T cells were stained with antibodies for CD4-FITC, CD69-PE, CD3ε-PE/Cy7, Foxp3-PerCP5.5, and CD44-PacificBlue (BioLegend, San Diego, CA). Intracellular staining for Foxp3-PerCP5.5 was performed using the classic protocol and reagents from eBioscience (San Diego,CA). Lung cells were also stained with B220-FITC, CD62L-PE, CD4-PEcy7, CD8b-PerCP5.5, CD3-AF700, and CD44-PB. Live and dead cells were labeled with LIVE/DEAD Fixable Aqua Dead Cell Stain Kit according to manufacturer’s instructions (Invitrogen by Life Technologies, Carlsbad, CA). Acquisition was done on a FACS Canto III (Becton Dickinson, Mountain View, CA) and analyzed used FlowJo software (Tree Star, Ashland, OR).

### Statistical Analysis

Prism 5 software (GraphPad Software, San Diego, CA) was used to perform statistical analysis. Statistical tests used included unpaired *t* tests, one-way ANOVA with the Bonferroni post hoc test between selected columns, and two-way ANOVA tests with the Bonferroni post hoc test. Statistically significant results were reported when *p* values were <0.05.

## Results

### Protocol 1: Re-Exposure Study

#### Allergic sensitization, inflammatory cell numbers, and AHR

**Figure 2:**
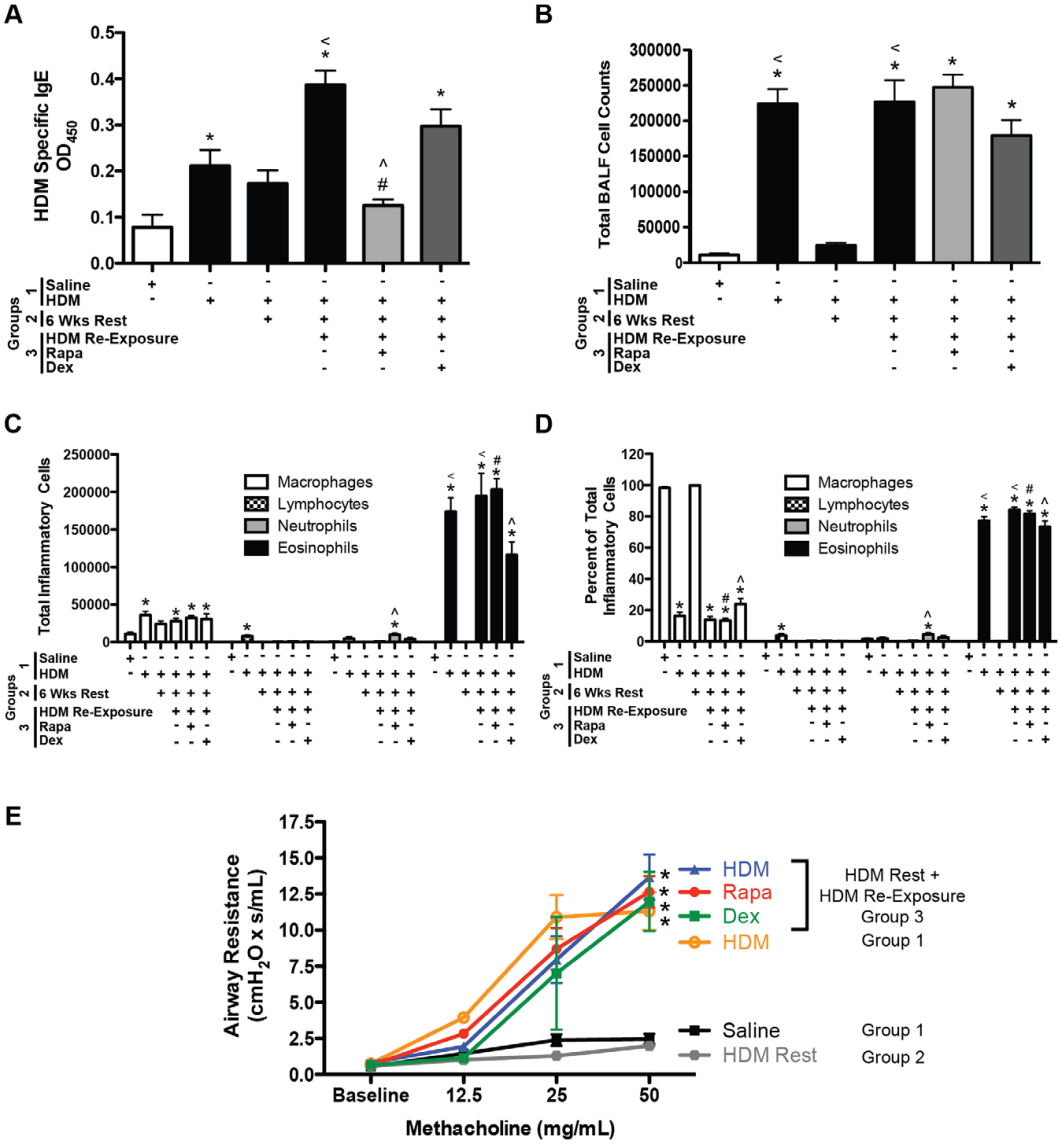
Protocol 1- Allergic sensitization, inflammatory cell numbers in the BALF, and AHR after HDM re-exposure. *A*, HDM-specific IgE levels were increased in HDM exposed (group 1) and HDM re-exposed (group 3) mice compared to saline controls. Rapamycin (Rapa) attenuated HDM-induced increases in IgE after allergen re-exposure, while dexamethasone (Dex) had no effect (*n = *4–12 mice/group). *p<0.05 versus saline; <*p*<0.05 versus HDM Rest; ∧*p*<0.05 versus HDM re-exposure; #*p*<0.05 versus Dex. *B*, Total BALF cell numbers were increased in HDM exposed mice in groups 1 and 3, but not in mice that rested for 6 weeks after HDM exposure (group 2) and unaltered by Rapa or Dex treatment (*n = *4–12 mice/group). *p<0.05 versus saline; <*p*<0.05 versus HDM Rest. *C,* Total numbers of macrophages and eosinophils were increased after HDM re-exposure (group 3), but not in HDM rest (group 2) animals in the BALF. Total neutrophil numbers in the BALF were slightly increased after HDM re-exposure in Rapa treated mice. Rapa did not suppress HDM-induced increases in eosinophils. Eosinophil numbers were lower in Dex treated mice compared to HDM re-exposed and Rapa treated groups, but still higher then saline control (*n = *4–12 mice/group). *p<0.05 versus saline; ∧*p*<0.05 versus HDM re-exposure; <*p*<0.05 versus HDM Rest; #*p*<0.05 versus Dex. *D*, The percentage of eosinophils in the BALF was increased in all HDM exposed groups except HDM rest mice, while the percentage of macrophages were reduced. (*n = *4–12 mice/group). *p<0.05 versus saline; ∧*p*<0.05 versus HDM re-exposure; <*p*<0.05 versus HDM Rest; #*p*<0.05 versus Dex. *E*, AHR was increased after the initial set of HDM exposures (group 1) compared to saline controls and was still increased after allergen re-exposure (group 3). Neither Rapa nor Dex suppressed HDM-induced increases in AHR after allergen re-exposure. AHR was similar to controls in HDM rest mice (group 2) (*n = *6–14 mice/group). *p<0.05 versus saline.

Allergic asthma is typically characterized by elevated IgE, eosinophilia and AHR. HDM-specific IgE levels were increased after the first round of HDM exposures (group 1) compared to saline controls (Figure 2A). After 6 weeks of rest/recovery (group 2), IgE levels were not increased compared to saline controls. To assess the impact of rapamycin and dexamethasone treatment on sensitization, HDM-specific IgE and HDM-specific IgG1 titers were assessed in the serum after HDM re-exposure (Figure 2A and Supplemental Figure 1A, respectively). Following allergen re-exposure (group 3), HDM-specific IgE levels increased further. Rapamycin treatment prevented this increase in IgE after HDM re-exposure, but dexamethasone treatment did not. HDM-specific IgG1 was increased in HDM exposed mice after 6 weeks of rest/recovery (group 2). There was no further increase in IgG1 after HDM re-exposure. Accordingly, neither rapamycin nor dexamethasone treatment suppressed HDM-specific IgG1 levels in the serum (group 3).

To assess inflammatory cell numbers, BALF was collected from the lungs. Total BALF cell numbers were increased after the first round of HDM exposures (group 1) (Figure 2B), most notably eosinophils (Figure 2C and D). After 6 weeks of rest (group 2), total BALF cell numbers, including lymphocytes, neutrophils, and eosinophils numbers were similar to saline controls (Figure 2B, C, and D). Mice re-exposed to HDM after 6 weeks of rest (group 3) demonstrated increases in total BALF cell numbers similar to those seen after the initial HDM exposure period (group 1) (Figure 2B) and this was mainly due to eosinophils (Figure 2C and D). Rapamycin treatment during HDM re-exposure did not suppress total BALF cell counts (Figure 2B). In particular, total eosinophil numbers and percentages remained elevated after rapamycin treatment (Figure 2C and D). There was a slight, but significant decrease in total BALF cell counts in the dexamethasone treated group that was mainly due to lower eosinophil numbers (Figure 2B, C, and D). Finally, AHR to methacholine was increased (50 mg/ml; 5.5 fold) in mice after the first round of HDM exposures (group 1) compared to saline controls (Figure 2E). In mice that rested/recovered for 6 weeks after HDM exposure (group 2), AHR was similar to saline controls. Upon allergen re-exposure (group 3), AHR increased in mice (50 mg/mL; 6 fold) compared to saline controls, but neither rapamycin nor dexamethasone treatment attenuated this increase in AHR (Figure 2E).

#### Goblet cells and airway remodeling

**Figure 3:**
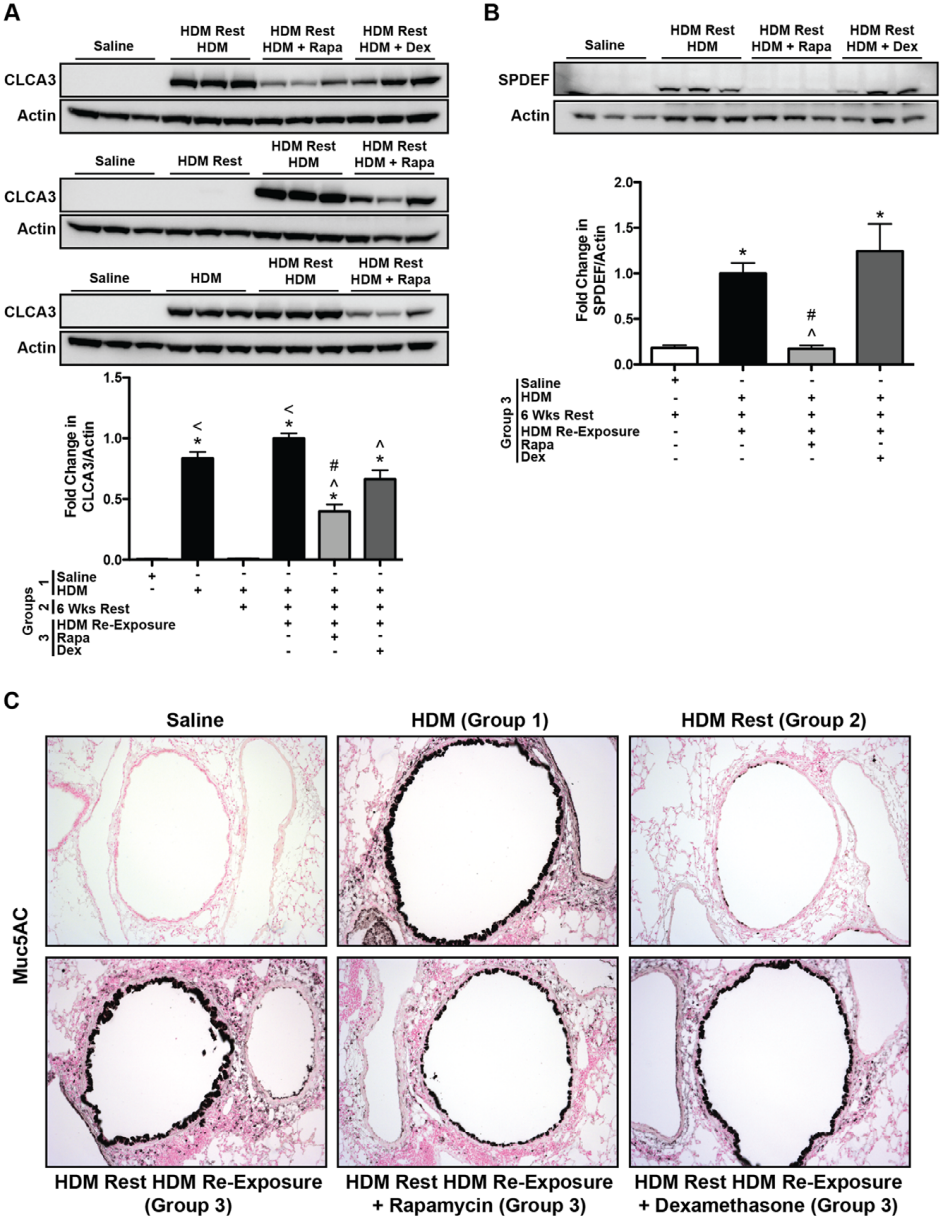
Protocol 1- Goblet cell markers in HDM re-exposed mice. *A*, Western blot analysis of lung homogenates showed that the goblet cell protein, CLCA3, was increased after the initial set of HDM exposures (group 1) and after HDM re-exposure (group 3). Both rapamycin (Rapa) and dexamethasone (Dex) treatment attenuated HDM-induced increases in CLCA3 (*n = *3–5 mice/group). *p<0.05 versus saline; *<p*<0.05 versus HDM Rest; ∧*p*<0.05 versus HDM re-exposed; #*p*<0.05 versus Dex. *B*, HDM-induced increases in the transcription factor, SPDEF, were suppressed by Rapa, but not Dex after allergen re-exposure (group 3). (*n = *3 mice/group). *p<0.05 versus saline; ∧*p*<0.05 versus HDM re-exposed; #*p*<0.05 versus Dex. *C*, Muc5AC immunostaining was increased in lung epithelial cells after HDM exposure (group 1) compared to saline controls, but was attenuated after 6 weeks of rest (group 2). After HDM re-exposure (Group 3), Muc5AC staining was increased again compared to saline controls and HDM rest (group 2). Rapamycin reduced Muc5AC staining in the lung, but staining was still elevated compared to saline controls. Dexamethasone treatment appeared to have minimal effects on HDM-induced increases in Muc5AC staining.

Another hallmark of asthma is increased airway mucus production. To assess changes in mucus producing goblet cells, the calcium-activated chloride channel 3 (CLCA3) protein, which associates with the mucin granule membranes of goblet cells [22], was measured in lung homogenates by Western blot analysis. In protocol 1, CLCA3 protein was not detectable in saline controls, but was readily detectable after the first round of HDM exposures (group 1) and with HDM re-exposure (group 3), but not after 6 weeks of rest (group 2) (Figure 3A). Rapamycin treatment attenuated increases in CLCA3 levels after HDM re-exposure, as did dexamethasone, although rapamycin was more effective. CLCA3 protein levels in both treatment groups were still higher than saline controls. Goblet cells also express the transcription factor SAM-pointed domain-containing Ets-like factor (SPDEF), which has been shown to be both necessary and sufficient for their differentiation into mucus producing goblet cells [25]. Similar to CLCA3, SPDEF levels were increased after HDM re-exposure (group 3) (Figure 3B). Rapamycin attenuated this increase, however, dexamethasone treatment did not suppress SPDEF levels (Figure 3B). In addition to CLCA3 and SPDEF, another goblet cell marker, Muc5AC, was also assessed. Immunohistochemical staining of lung sections showed increases in Muc5AC staining in the epithelial cells of HDM exposed mice (group 1) compared to saline control, that was attenuated after 6 weeks of rest (group 2) (Figure 3C). Muc5AC staining increased again after HDM re-exposure (group 3), but staining was reduced with rapamycin treatment (Figure 3C). Only a slight reduction in Muc5AC staining was observed in dexamethasone treated mice (Figure 3C). In addition to Muc5AC, we also performed immunohistochemical staining for the smooth muscle cell marker, α-smooth muscle actin (α-SMA), to assess airway muscularization and remodeling. Similar levels of α-SMA staining were observed between all groups (Supplemental Figure 5A). This is consistent with there being no differences in baseline airway resistance between the groups (Figure 2E).

**Figure 5:**
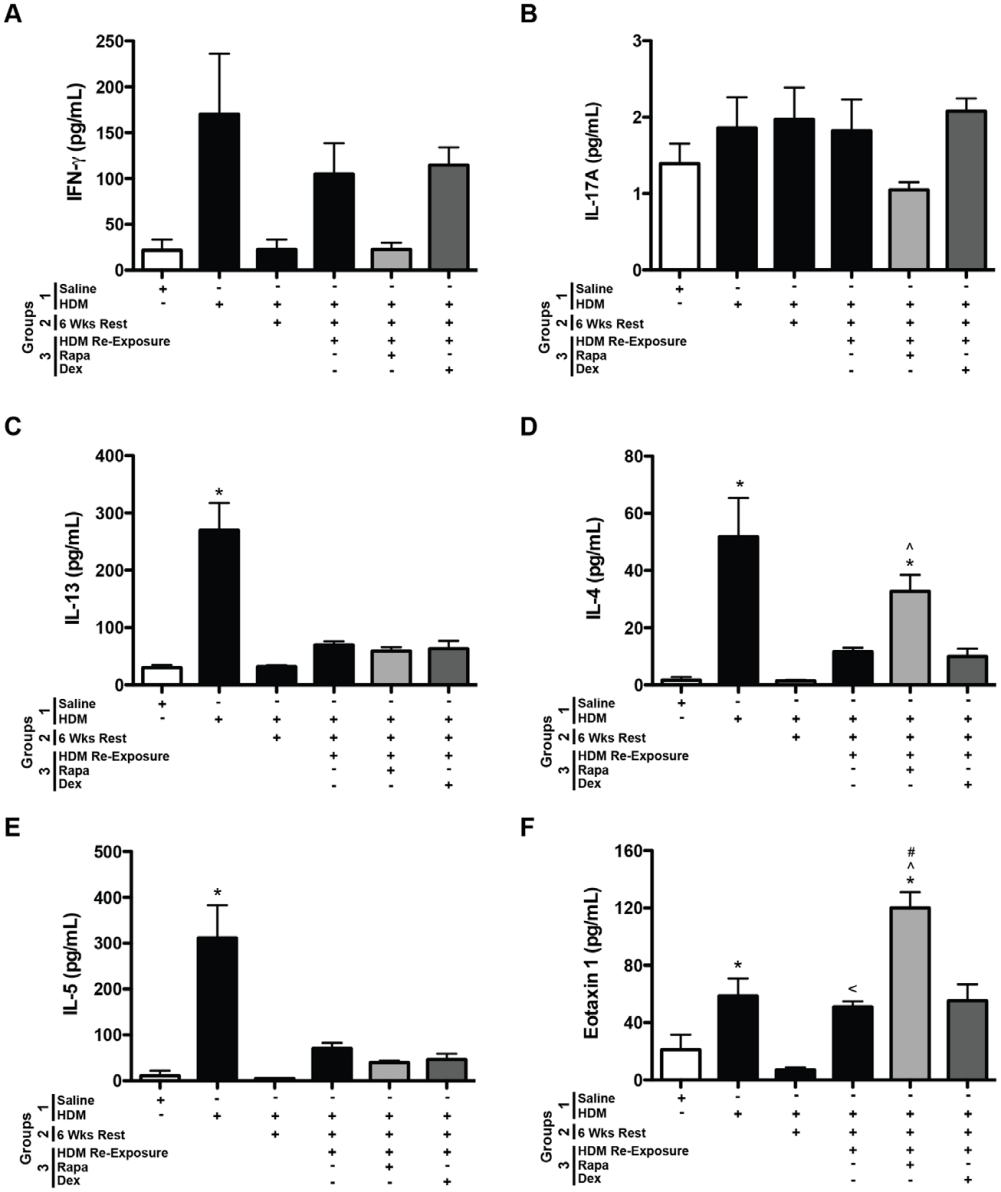
Protocol 1- Th1, Th2, and Th17 cytokines and chemokines in BALF of HDM re-exposed mice. *A,* Levels of INF-γ appeared to be lower after rapamycin (Rapa) treatment in HDM re-exposed mice, but were not statistically different between any of the groups (*n = *3–7 mice/group). *B*, IL-17A levels in the BALF were not different between groups (*n = *3–8 mice/group). *C*, IL-13 levels were increased after the initial set of HDM exposures (group 1), but not after allergen re-exposure (group 3). Neither Rapa nor dexamethasone (Dex) had any effect on IL-13 levels after HDM re-exposure (*n = *3–8 mice/group). *D*, IL-4 levels were increased after the first set of HDM exposures (group 1), but not after HDM re-exposure (group 3). Rapa treatment augmented IL-4 levels after HDM re-exposure, but Dex did not. *E*, IL-5 levels were increased in group 1 after the initial set of HDM exposures, but not after HDM re-exposure (group 3). IL-5 levels were unaffected by Rapa or Dex treatment following HDM re-exposure (*n = *3–8 mice/group). *p<0.05 versus saline. *F*, Eotaxin 1 levels were increased in group 1 after HDM exposure. After HDM re-exposure, Rapa augmented eotaxin 1 levels (*n = *3–8 mice/group). *p<0.05 versus saline; <*p*<0.05 versus HDM Rest; ∧*p*<0.05 versus HDM re-exposed; #*p*<0.05 versus Dex.

#### Systemic and local cellularity

HDM re-exposure (group 3) induced an increase in lung tissue cellular infiltrate (Supplemental Figure 2B), associated with increased spleen weights (Supplemental Figure 2A). Rapamycin and dexamethasone treatments both lead to decreases in spleen weights and lung cellularity (Supplemental Figure 2A and 2B) indicating broad anti-inflammatory impact. To specifically address the impact of these treatments on lung lymphocytes, flow cytometry was used to identify B and T cells. Lung CD3^+^ T cells numbers were increased following HDM re-exposure (Supplemental Figure 2C). Both rapamycin and dexamethasone treatment abrogated this influx of T cells into the lung tissue (Supplemental Figure 2C). A trend towards a decrease in the number of B cells after rapamycin treatment was observed; however, this did not reach statistical significance (Supplemental Figure 2D).

#### Lung T cells

**Figure 4:**
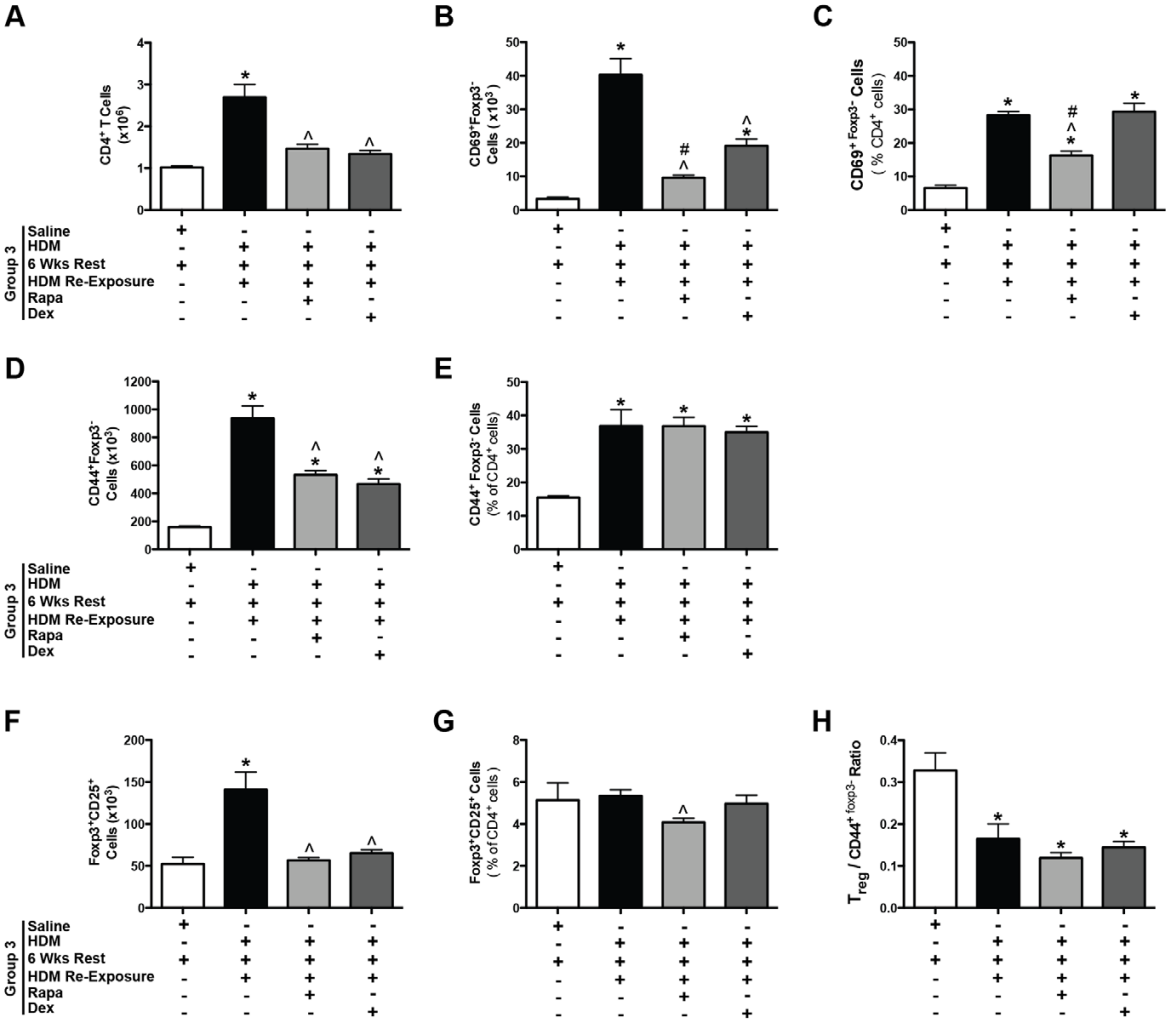
Protocol 1- T cell populations in mice after HDM re-exposure. *A*, HDM-induced increases in total CD4^+^ lung cells after allergen re-exposure were suppressed by rapamycin (Rapa) and dexamethasone (Dex) (*n = *4–12 mice/group). *p<0.05 versus saline; ∧*p*<0.05 versus HDM re-exposed. *B*, Activated T cells (CD69^+^Foxp3^−^) were increased after HDM re-exposure and suppressed by Rapa and Dex (*n* = 4–12 mice/group). *p<0.05 versus saline; ∧*p*<0.05 versus HDM re-exposed; #*p<*0.05 versus dex. *C*, CD69^+^Foxp3^−^ activated T cells, as a percentage of total CD4^+^ T cells was also increased after HDM re-exposure and suppressed by Rapa, but not Dex (*n = *4–12 mice/group) *p<0.05 versus saline; ∧*p*<0.05 versus HDM re-exposed; #*p<*0.05 versus dex. *D,* Lung effector T cells (CD44^+^Foxp3^−^) were increased after HDM re-exposure and attenuated by Rapa and Dex (*n = *4–12 mice/group). *p<0.05 versus saline; ∧*p*<0.05 versus HDM re-exposed. *E,* CD44^+^Foxp3^−^ effector cells, as a percentage of total CD4^+^ T cells were increased in all HDM re-exposed groups and not suppressed by Rapa or Dex (*n* = 4–12 mice/group). *p<0.05 versus saline. *F*, Total lung regulatory T cells (Foxp3^+^CD25^+^) were increased after HDM re-exposure and suppressed by Rapa and Dex (*n = *4–12 mice/group). *p<0.05 versus saline; ∧*p*<0.05 versus HDM re-exposed. *G*, Foxp3^+^CD25^+^cells, as a percentage of total lung CD4^+^ T cells, were slightly reduced by Rapa treatment, but not Dex (*n = *4–12 mice/group). ∧*p*<0.05 versus HDM re-exposed. *H*, The ratio of Foxp3^+^CD25^+^ regulatory T cells/CD44^+^Foxp3^−^ effector T cells was lower in HDM re-exposed mice compared to saline controls, as well as Rapa and Dex groups (*n* = 4–12 mice/group). *p<0.05 versus saline.

Since mTOR has previously been shown to play an important role in the growth and proliferation of T cells, we focused on the effects of rapamycin and dexamethasone on CD4^+^ T cells. In this study, we characterized T cell subsets after mice were treated with HDM, rested/recovered for 6 weeks, and then were re-exposed to HDM since our earlier studies [18] had already assessed T cells in the early stages of HDM-induced allergic airway disease. Similar to total CD3^+^ lung cells, total CD4^+^ lung T cells were increased after HDM re-exposure (group 3) compared to saline controls (Figure 4A). Both rapamycin and dexamethasone treatment suppressed this increase. Next we assessed specific T cell populations in the lung tissue. CD69^+^ activated T cells were increased after HDM re-exposure compared to saline controls (Figure 4B). Both rapamycin and dexamethasone suppressed this response although rapamycin treatment was more effective since the number of CD69^+^ T cells were still increased in dexamethasone treated mice compared to saline controls (Figure 4B and Supplemental Figure 7A). When CD69^+^ activated T cells were assessed as a percentage of total CD4^+^ T cells, all HDM re-exposed mice showed increases in the percentage of CD69^+^ T cells compared to saline controls, but only rapamycin treatment suppressed this response compared to HDM alone (Figure 4C). CD44^+^ effector T cells were also increased after HDM re-exposure compared to saline controls (Figure 4D). Rapamycin and dexamethasone suppressed this response, however, the number of effector T cells was still increased in these groups compared to saline controls. When the percentage of CD44^+^ effector T cells was determined, increases were observed in all HDM re-exposed mice, but neither rapamycin nor dexamethasone suppressed this response (Figure 4E). Total Foxp3^+^CD25^+^ regulatory T cell numbers were also increased in HDM re-exposed mice compared to saline controls (Figure 4F) and were completely suppressed by rapamycin and dexamethasone treatment. When regulatory T cells were assessed as a percentage of CD4^+^ T cells, rapamycin, but not dexamethasone, suppressed regulatory T cells, (Figure 4G). Finally, the ratio of regulatory T cells to CD44^+^ effector T cells was also determined and all HDM exposed mice demonstrated decreased ratios compared to saline controls (Figure 4H). Taken together, these results suggest that rapamycin and dexamethasone treatment decrease many lung T cell populations, however, it is unclear if the effects of these drugs are specific to T cells since both drugs also decreased total lung cells (Supplemental Figure 2B). In addition, there was a trend towards a decrease in the number of B cells after rapamycin treatment; however, this did not reach statistical significance (Supplemental Figure 2D).

**Figure 7:**
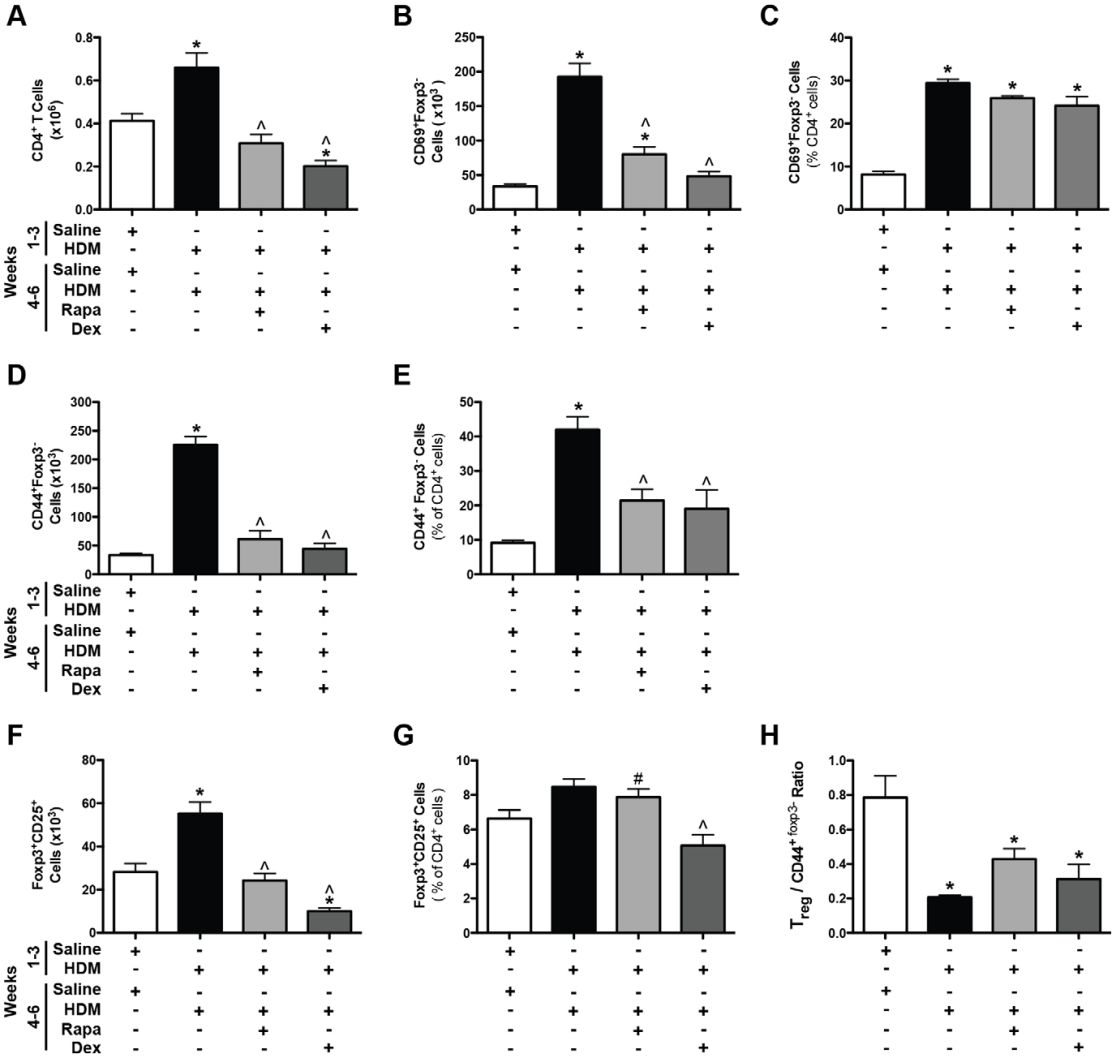
Protocol 2- T cell populations after chronic HDM exposure. *A,* Total CD4^+^ T cells were increased after 6 weeks of HDM and suppressed by rapamycin (Rapa) and dexamethasone (Dex) (*n = *4–12 mice/group). *p<0.05 versus saline; ∧*p*<0.05 versus HDM. *B*, Total activated T cells (CD69^+^Foxp3^−^) were increased in mice after 6 weeks of chronic HDM exposure and suppressed by Rapa and Dex (*n = *6–8 mice/group). *p<0.05 versus saline; ∧*p*<0.05 versus HDM. *C,* CD69^+^Foxp3^−^ activated T cells, when assessed as a percentage of total CD4^+^ T cells, were increased after HDM exposure and unaffected by Rapa and Dex (*n = *6–8 mice/group). *p<0.05 versus saline. *D*, Total effector T cells (CD44^+^Foxp3^−^) were increased after 6 weeks of HDM exposure and suppressed by Rapa and Dex (*n = *6–8 mice/group). *p<0.05 versus saline; ∧*p*<0.05 versus HDM. *E,* CD44^+^Foxp3^−^ effector T cells, when expressed as a percentage of total CD4^+^ T cells, were increased after HDM exposure and attenuated by Rapa and Dex (*n = *6–8 mice/group). *p<0.05 versus saline; ∧*p*<0.05 versus HDM. *F,* Total lung regulatory T cells (Foxp3^+^CD25^+^) were increased after chronic HDM exposure and suppressed by Rapa and Dex (*n = *6–8 mice/group). *p<0.05 versus saline; ∧*p*<0.05 versus HDM. *G*, Foxp3^+^CD25^+^ T cells, as a percentage of total lung CD4^+^ T cells, were reduced by Dex, but not by Rapa (*n = *6–8 mice/group). ∧*p*<0.05 versus HDM;^ #^ *p*<0.05 versus Rapa. *H,* The ratio of regulatory T cells Foxp3^+^CD25^+^ to CD44^+^Foxp3^−^ effector T cells was decreased in HDM exposed mice compared to saline controls (*n = *6–8 mice/group). *p<0.05 versus saline.

#### Cytokines

Cytokines were assessed to identify mediators of the allergic responses. Since mTOR inhibition has been previously shown to affect T cell differentiation [11], [12], [13], Th1, Th2, and Th17 cytokines were assessed in the BALF. After HDM re-exposure, a trend for increased INF-γ, a Th1 cytokine, was observed in HDM exposed mice, but none of the groups were significantly increased compared to saline controls (Figure 5A). Similarly, although the IFN-γ response appeared lower in the rapamycin treated group, these data were not significant. The levels of the Th17 cytokine, IL-17A, were not significantly increased at this time point in any HDM exposed mice compared to saline controls (Figure 5B). IL-17A levels were lower with rapamycin treatment; however, this was not statistically different from HDM re-exposed mice. Th2 cytokines, IL-4, IL-5, IL-13 and the chemokine, eotaxin 1, were all increased in BALF after the first round of HDM exposures (group 1) compared to saline controls (Figure 5C–F). All of these cytokines returned to saline control levels after 6 weeks of rest (group 2). After HDM re-exposure, IL-4, IL-5, IL-13, and eotaxin 1 levels were slightly higher, however, these values did not reach statistical significance compared to saline controls. Neither rapamycin nor dexamethasone had any suppressive effects on the levels of these mediators. In fact, rapamycin treatment during HDM re-exposure augmented the IL-4 and eotaxin 1 levels compared to saline controls and mice re-exposed to HDM only (Figure 5D and F).

#### mTOR signaling: P-S6 and P-Akt

**Figure 6:**
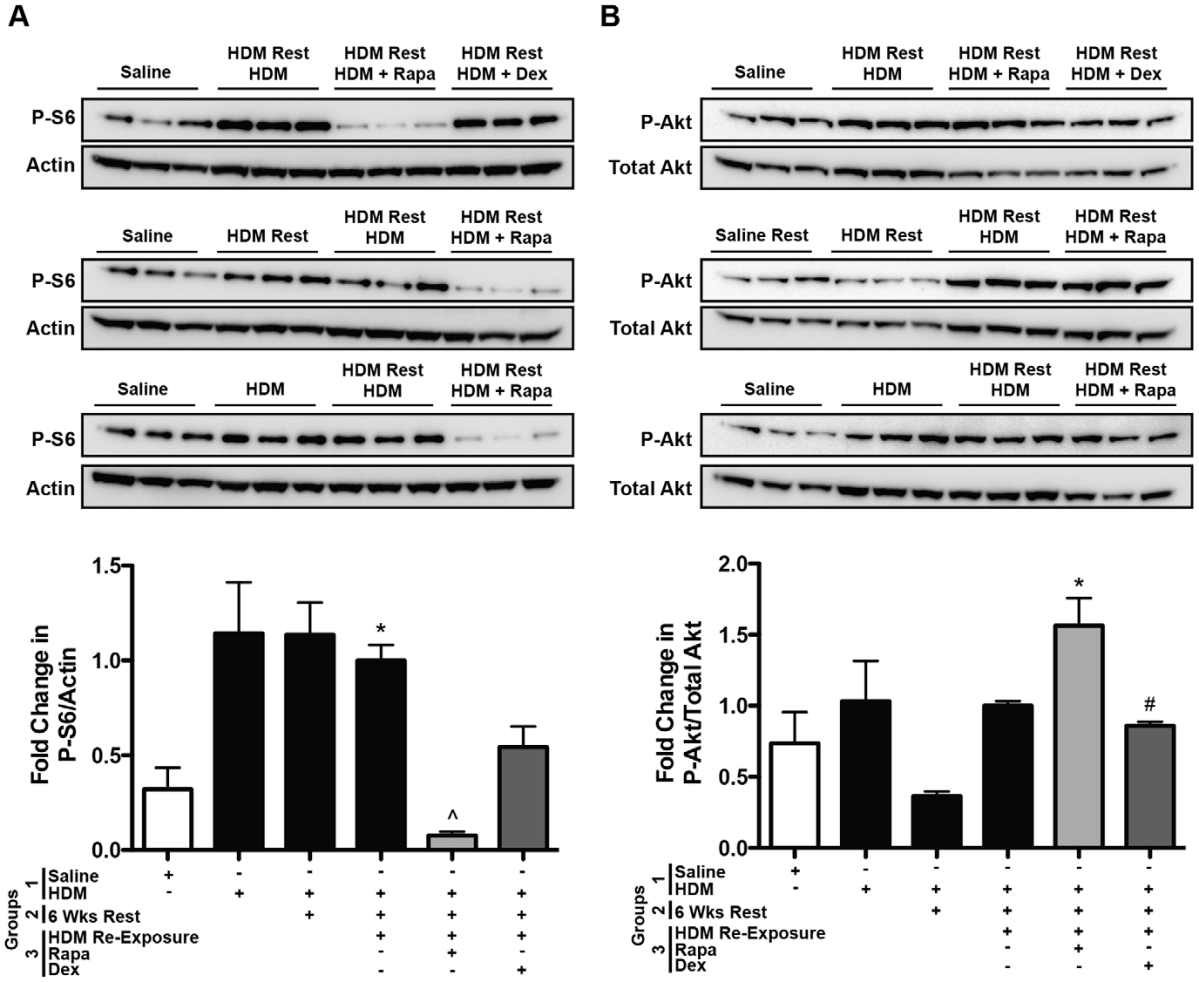
Protocol 1- Western blot of P-S6 and P-Akt in lung homogenates of HDM re-exposed mice. *A,* P-S6, a downstream mediator of mTOR complex 1 signaling, was increased in HDM re-exposed mice (group 3) and this was blocked by rapamycin (Rapa) treatment (*n = *3–5 mice/group). *p<0.05 versus saline; ∧*p*<0.05 versus HDM re-exposed. *B*, P-Akt, a downstream mediator of mTOR complex 2 signaling, was increased after allergen re-exposure in Rapa treated mice compared to saline controls (*n = *3–5 mice/group), but unaffected by Dex. *p<0.05 versus saline; #*p*<0.05 versus Rapa.

Phosphorylation of S6, which is downstream of the rapamycin sensitive mTOR complex 1 (mTORC1), was measured by Western blot analysis on lung homogenates to determine whether the dose of rapamycin used in these studies was sufficient since rapamycin treatment had limited effects on the asthmatic response during allergen re-exposure. Phosphorylation of S6 was increased in HDM re-exposed mice (group 3) compared to saline controls (Figure 6A). This increase was completely blocked by rapamycin treatment, but not dexamethasone. Phosphorylation of Akt (S473), which is downstream of mTOR complex 2 (mTORC2), was not suppressed by rapamycin treatment (Figure 6B). These data suggest that the dose of rapamycin used in this study was sufficient to block the mTORC1 pathway, but not mTORC2.

### Protocol 2: Reversal Study

#### Allergic sensitization, inflammatory cell numbers, and AHR

Allergic sensitization was assessed by measuring HDM-specific IgG1 and IgE levels in the serum of mice exposed to HDM intranasally, 3 times a week for either 3 or 6 weeks. HDM-specific IgG1 levels were increased after both 3 and 6 weeks of HDM (Supplemental Figure 1B). Both rapamycin and dexamethasone treatment attenuated these increases in HDM-specific IgG1, but levels were still increased compared to saline controls. HDM-specific IgE levels were also increased in mice exposed to HDM for 3 and 6 weeks (Supplemental Figure 3A), and reduced by rapamycin and dexamethasone compared to mice exposed to HDM alone for 3 or 6 weeks. Total BALF cell numbers were increased after 3 and 6 weeks of HDM exposure compared to saline controls (Supplemental Figure 3B). In rapamycin treated mice, total BALF cell numbers were higher compared to mice exposed to HDM alone for 6 weeks. The augmented total BALF cell numbers in rapamycin treated mice were mainly due to an increase in macrophages and eosinophils (Supplemental Figure 3C and D). Dexamethasone treatment suppressed total BALF cell numbers compared to mice exposed to HDM alone for 6 weeks and this was mainly due to a decrease in eosinophils (Supplemental Figure 3C and D). AHR was increased in all HDM exposed groups at 50 mg/ml methacholine compared to saline controls. Neither rapamycin nor dexamethasone treatment attenuated HDM-induced AHR (Supplemental Figure 3E) at 50 mg/ml; however, dexamethasone did suppress AHR at 25 mg/ml compared to mice exposed to HDM for 3 or 6 weeks.

#### Goblet cells and airway remodeling

CLCA3 protein levels in the lung were barely detectable in saline controls (Supplemental Figure 4A), but were increased after 3 and 6 weeks of chronic HDM exposure compared to controls. CLCA3 protein levels were not reduced by rapamycin, but were reduced by dexamethasone. SPDEF protein levels were increased in mice exposed to HDM for 6 weeks. SPDEF levels were not altered with rapamycin treatment and while there was a trend for lower SPDEF levels with dexamethasone, these levels did not reach statistical significance (Supplemental Figure 4B). Muc5AC staining of lung tissue was also increased after both 3 and 6 weeks of HDM exposure compared to saline controls (Supplemental Figure 4C). Muc5AC staining was similar between mice exposed to HDM alone for 6 weeks and mice treated with rapamycin. Muc5AC staining appeared attenuated with dexamethasone treatment. When airway muscularization and remodeling was assessed by α-SMA immunohistochemistry staining, no noticeable differences between the animal groups were observed (Supplemental Figure 5B). This is consistent with there being no differences in baseline airway resistance between the groups (Supplemental Figure 3E).

#### Lung T Cells

Total lung CD4^+^ T cells were increased after 6 weeks of HDM exposure compared to saline controls, and both rapamycin and dexamethasone treatment suppressed this response (Figure 7A). Next, different T cell populations were assessed in the lung. Total CD69^+^ activated CD4^+^ T cells were increased after 6 weeks of HDM exposures compared to saline controls (Figure 7B). Rapamycin and dexamethasone treatment suppressed this response. When CD69^+^ activated T cells were measured as a percentage of total CD4^+^ T cells, animals exposed to HDM for 6 weeks showed an increase in the percentage of activated T cells compared to saline controls (Figure 7C and Supplemental Figure 7B). However, rapamycin and dexamethasone had a limited impact on the percentage of activated T cells (Figure 7C and Supplemental Figure 7B). Total CD44^+^ effector T cells were also determined and were increased after HDM exposure and decreased after rapamycin and dexamethasone treatment (Figure 7D). Similarly, when CD44^+^ effector cells were assessed as a percentage of total CD4^+^ T cells, effector T cells percentages were increased with HDM exposure and partially decreased by rapamycin and dexamethasone (Figure 7E). Total Foxp3^+^CD25^+^ regulatory T cell numbers were also increased in HDM exposed mice compared to saline controls (Figure 7F), and both rapamycin and dexamethasone suppressed this response. When regulatory T cells were assessed as a percentage of all CD4^+^ T cells, only dexamethasone suppressed regulatory T cells (Figure 7G). Finally, the ratio of regulatory T cells to CD44^+^ effector T cells was also determined and was decreased in all HDM exposed mice (Figure 7H). Although rapamycin and dexamethasone appeared to have specific suppressive effects on different T cell populations in the lung tissue, spleen weights (Supplemental Figure 2E) were also decreased by both drugs, suggesting that the effects of rapamycin and dexamethasone may not be specific to T cells. Finally, dexamethasone suppressed total lung cells and B cells and although there was a trend towards a decrease in B cells after rapamycin treatment, this did not reach statistical significance (Supplemental Figure 2F and H).

#### Cytokines

**Figure 8:**
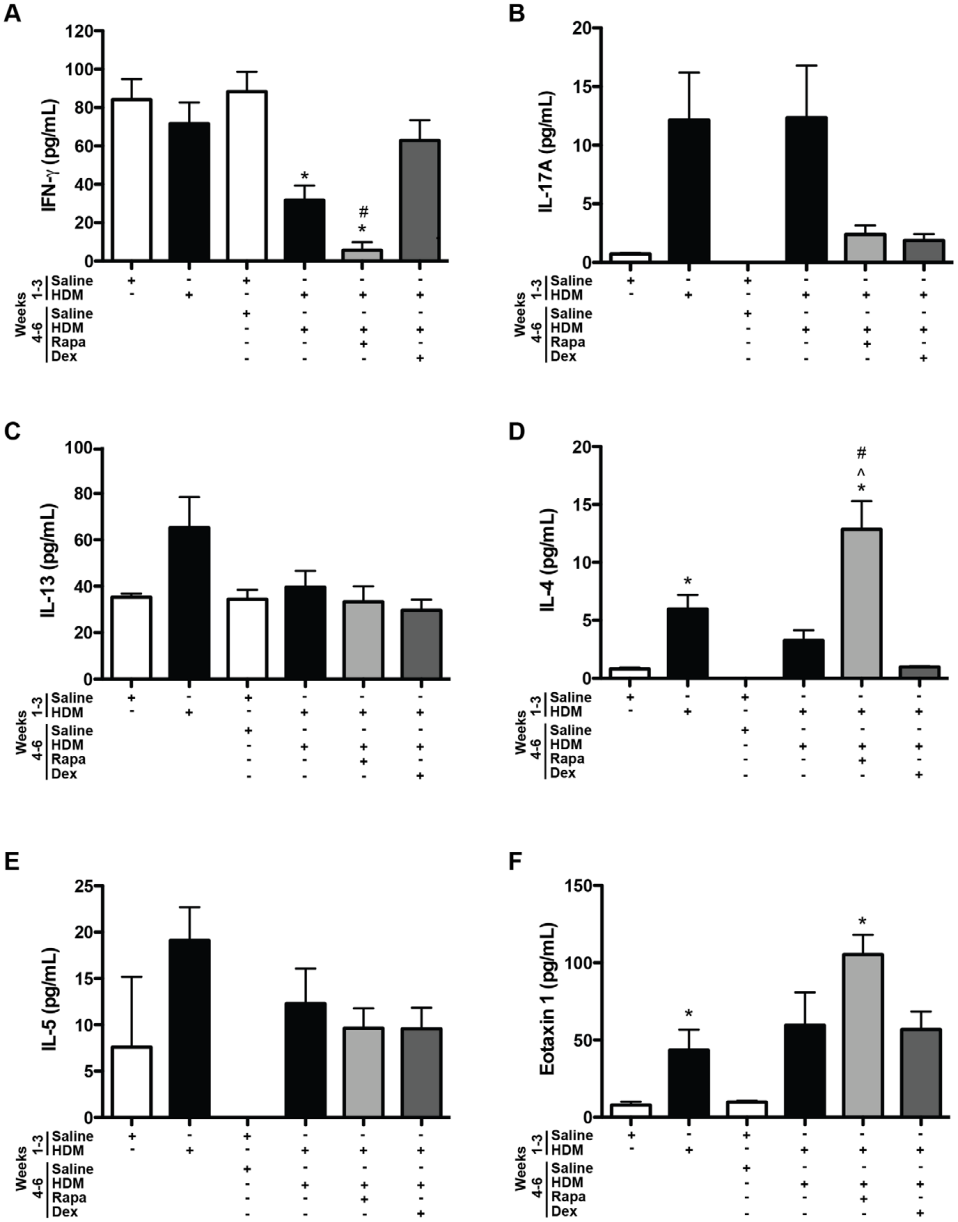
Protocol 2- Th1, Th2, and Th17 cytokines and chemokines in the BALF after chronic HDM exposure. *A*, INF-γ was suppressed after 6 weeks of HDM exposure and in the rapamycin (Rapa) treated HDM group (*n = *3–5 mice/group). *p<0.05 versus saline; ^#^ *p*<0.05 versus Dex. *B*, No significant differences in IL-17A levels were observed between animal groups, although there were trends for increased IL-17 in the HDM exposed group and lower leves in mice exposed to HDM and treated with Rapa or dexamethasone (Dex) during weeks 4–6 (*n = *3–5 mice/group). *C*, IL-13 levels were not significantly altered with HDM exposure, by Rapa or by Dex treatment in this model (*n = *3–5 mice/group). *D*, IL-4 levels were increased after 3 weeks of HDM compared to saline controls. After 6 weeks of HDM, IL-4 levels were higher in the Rapa treated group. IL-4 levels were similar between HDM and Dex treated mice (*n = *3–5 mice/group). *p<0.05 versus saline; ∧*p*<0.05 versus HDM; ^#^ *p*<0.05 versus Dex. *E*, No statistically significant differences in IL-5 levels were observed between any group of mice (*n = *3–5 mice/group). *F*, Eotaxin 1 levels were increased after 3 weeks of HDM exposure and were higher in the Rapa treated group after 6 weeks of chronic HDM compared to saline controls (*n = *3–5 mice/group). *p<0.05 versus saline.

IFN-γ levels in the BALF were lower after 6 weeks of HDM and rapamycin treatment, but unaffected by dexamethasone (Figure 8A). IL-17A levels appeared to be increased after 3 and 6 weeks of HDM exposure and lower with rapamycin and dexamethasone treatment; however, none of the differences reached statistical significance (Figure 8B). Although there was an upward trend, neither IL-13 nor IL-5 levels were significantly increased in the BALF after 3 weeks of HDM exposure (Figure 8 C and E). IL-4 and eotaxin 1 levels were significantly increased after 3 weeks of HDM exposure (Figure 8D and F). By six weeks of HDM exposure, although there were upward trends for increases in IL-4, IL-5, and eotaxin 1 levels, they were not significantly increased in mice compared to saline controls. Interestingly, IL-4 and eotaxin 1 levels were augmented with rapamycin treatment compared to saline controls (Figure 8 D and F). Th2 cytokine levels were unaltered with dexamethasone treatment at this time point.

#### mTOR signaling: P-S6 and P-Akt

Similar to the findings in the re-exposure study, Western blot analysis demonstrated increases in phosphorylated S6 (P-S6) in lung tissue after 3 weeks of HDM exposure (Supplemental Figure 6A). P-S6 levels were also increased after 6 weeks of HDM exposure and suppressed by rapamycin and dexamethasone treatment. Phosphorylated Akt (S473) levels were unaltered by rapamycin treatment (Supplemental Figure 6B). These data indicate that the dose of rapamycin used in this study was sufficient to block mTORC1 signaling, but not mTORC2.

## Discussion

The goal of our study was to determine whether mTOR inhibition with rapamycin would suppress the key features and mediators of HDM-induced allergic asthma in established asthmatic disease. In addition, we also compared rapamycin to the steroid, dexamethasone, since steroids are currently a mainstay treatment for asthma. In the first protocol, we assessed whether rapamycin or dexamethasone could suppress allergic disease during allergen re-exposure. Although rapamycin suppressed IgE levels, goblet cells, and total lung T cells, it had no effect on AHR or BALF cellularity and IL-4 and eotaxin 1 levels were actually augmented. Dexamethasone suppressed goblet cells and total lung T cells, but had no effect on IgE or AHR and only slightly reduced BALF eosinophilia. Our second protocol assessed whether rapamycin or dexamethasone could reverse or inhibit the progression of asthmatic responses during chronic allergic airway disease. In this model, rapamycin did not suppress AHR or goblet cells and actually augmented inflammatory cell numbers, IL-4 and eotaxin 1 levels in the BALF. Dexamethasone had limited effects on AHR, but did attenuate the inflammatory cell influx into the BALF, especially eosinophils. Despite these limited effects, both rapamycin and dexamethasone suppressed lung tissue lymphocyte numbers and serum IgE levels.

Previous studies from our lab demonstrated that HDM-induced allergic asthma could be prevented if rapamycin was administered early and simultaneously with HDM. In this case, rapamycin prevented HDM-induced AHR, inflammation, goblet cells, and allergic sensitization [18]. Since allergic sensitization was suppressed in our previous studies, we also determined whether rapamycin could prevent allergic responses once sensitization had already been established. To do this, mice were first sensitized systemically to HDM by i.p. injection. During subsequent intranasal HDM exposures, mice were treated with rapamycin. In this case, rapamycin still suppressed many of the key allergic responses including IgE, AHR, goblet cells, T cell responses, and key mediators like IL-13 and leukotrienes, although it did not suppress increases in inflammatory cells in the BALF [18], which may have partly been due to the chemokine, eotaxin 1, since levels were still elevated after rapamycin treatment. Although these studies demonstrated an important role for the mTOR pathway during early allergic sensitization and asthmatic disease processes, it was unclear whether mTOR signaling would be important during allergen re-exposure or during established/progressive allergic disease. The studies we report in this manuscript sought to address this question. These data suggests that the role of mTOR is very different depending on the timing/disease stage since rapamycin treatment during allergen re-exposure or during chronic, ongoing disease did not attenuate key characteristics of allergic asthma including AHR and inflammation and actually augmented IL-4 and eotaxin 1 levels. The results from our second protocol are similar to that of a recent study published by Fredriksson et. al. who demonstrated that rapamycin did not suppress allergic responses when administered during chronic allergic disease [26]. In addition, our studies demonstrated that rapamycin suppressed T cells in the lung tissue, including regulatory T cells and our studies also compared the effects of rapamycin to the steroid, dexamethasone.

Allergic asthma is often treated with steroids to suppress inflammation [1], [27]. Previous studies have utilized the corticosteroid, dexamethasone, in allergic asthma models [28], [29], [30]. For example, a study similar to ours investigated the effects of dexamethasone during allergic relapse and overt disease. In an OVA model of allergic airway disease, dexamethasone suppressed goblet cells, serum IgE, AHR, and reduced airway inflammation in a relapse model. During overt disease, dexamethasone reduced goblet cells, AHR, and the number of eosinophils, but had no effect on serum IgE levels [28]. In our HDM-induced model of allergen re-exposure/relapse, dexamethasone also decreased goblet cells, but did not suppress IgE or AHR and eosinophil numbers were only slightly reduced. Likewise, during chronic, ongoing or overt disease, we did not observe suppression of goblet cells and the effects on AHR were limited, although there were decreases in inflammatory cell numbers, specifically eosinophils. Although the decrease in eosinophils in this study was as expected with dexamethasone treatment, no decrease in AHR was surprising. However, previous reports have suggested that the timing of AHR measurements after dexamethasone treatment may be important [29]. Specifically, when AHR was measured 12 hours after dexamethasone treatment, AHR was suppressed, but by 24 hours after dexamethasone treatment, AHR was no longer suppressed [29]. In all of our studies, AHR was assessed 24 hours after the last dexamethasone treatment, which may explain why limited decreases in AHR were observed. In addition, differences between the results of our study and others may be due to the type of allergen used and/or the route of dexamethasone treatment since ultrasonic nebulization was used in the study by Jungsuwadee et. al. [28], versus i.p. delivery in our study.

Unlike our previous studies when rapamycin was given early in the disease process [18], in this study, AHR was not suppressed by rapamycin during allergen re-exposure or chronic allergic disease. Both IL-4 and inflammation were still increased after rapamycin treatment and could potentially contribute to AHR [31]. However, in our previous study, rapamycin treatment decreased AHR despite elevated IL-4 BALF levels and inflammatory cell numbers suggesting that other mechanisms are involved [18]. IL-13 is a key mediator of AHR, however, in these chronic/established models, IL-13 was not increased, suggesting that it may not be contributing to AHR in more advanced disease. Airway remodeling is another feature of asthma that could contribute to sustained AHR in these more advanced disease models; however, no major differences in airway smooth muscle or baseline airway resistance between animal groups in either study were observed, suggesting other mechanisms are playing a role in the disease process.

An interesting observation in our studies was that IL-4 levels were higher even though IgE levels after HDM exposure were still suppressed by rapamycin treatment. This was surprising since IL-4 is an important mediator of IgE class switching. It is possible that rapamycin could directly affect B cells that are secreting IgE to suppress allergic sensitization to HDM. Previous work has demonstrated that mTOR is required for B cell development and maturation [32], [33], however, less is known about the role of mTOR in B cell homeostasis, activation of mature B cells, and immunoglobulin production/secretion. A study using purified human B cells demonstrated that rapamycin inhibited B cell proliferation, induced apoptosis, and suppressed immunoglobulin production, particularly IgM and IgG [34]. Although these studies were carried out *in vitro* they still suggest that rapamycin could have direct effects on B cells, which could account for the decreases in IgE levels in our *in vivo* model and therefore reduce sensitization to HDM, despite increased IL-4. When we assessed B cells in the lung tissue, there was a trend towards a decrease in B cells in both studies after rapamycin treatment. Despite being non-significant, we cannot exclude that this minor decrease in lung B cell levels could contribute to the observed decrease in IgE levels. The source of the IL-4 increase is unclear in our model since T cells, which are one of the primary sources of IL-4, were reduced. Other cells including eosinophils, basophils, and mast cells can secrete IL-4 [35], but whether these cells are playing a role in enhancing IL-4 levels in our model is unclear. Also in our study, eotaxin 1, an important epithelial cell derived eosinophil chemokine, remained elevated in the BALF with rapamycin treatment, which may explain why eosinophil numbers were not suppressed. This was also true in our previous acute study in which rapamycin treatment did not suppress airway inflammation nor eotaxin 1 levels once sensitization was established [18].

More recent studies have indicated an important role for regulatory T cells in the resolution of allergic airway disease [36], [37], [38]. Studies have demonstrated that adoptive transfer of CD4^+^CD25^+^Foxp3^+^ regulatory T cells into mice exposed to allergen suppressed allergic responses, whereas inhibition of regulatory T cells exacerbated the allergic response [39]. *In vitro* data suggests that rapamycin can expand CD4^+^CD25^+^Foxp3^+^ regulatory T cells in the presence of IL-2 [40], [41], however, in our *in vivo* model, rapamycin treatment was associated with decreases in effector T cells, a major source of IL-2 in the lung. Hence, rapamycin treatment, much like dexamethasone treatment, may decrease regulatory T cells *in vivo* by decreasing the number of IL-2 producing cells. It is unclear if the reductions in regulatory T cells after rapamycin treatment in this model would have any biological significance; however, loss of regulatory T cells has been shown to worsen the severity of allergic disease [42]. Interestingly, loss of CD69^+^ cells has also been associated with exacerbated allergic disease [43]. These findings remain controversial however [44], as new roles for CD69 in cell egress from lymphoid organs, Th17 differentiation and formation of memory CD44^+^CD4^+^ T cells are being proposed [45], [46], [47]. Interestingly, short rapamycin (or dexamethasone) treatment had little effect on the proportion of memory cells in the lungs, whereas longer exposure to rapamycin (or dexamethasone) in our second model significantly decreased the proportion of CD44^+^ memory cells among CD4^+^ T cells.

It remains unclear why many of the allergic responses, especially AHR, were not suppressed in our studies even though T cell populations were reduced. The effects of rapamycin and dexamethasone, however, may not be only specific to T cells since spleen sizes were also reduced in our studies, consistent with the immunosuppressive properties of these drugs [48], [49]. It is possible that other cell types in the lung could be contributing to the allergic responses in these established/chronic models, uch as epithelial cells. Epithelial cells and other lung cells can produce cytokines upon allergen exposure, which can then directly influence allergic responses, including AHR [50], [51].

The protein encoded by the mTOR gene signals through two protein complexes, mTOR complex 1 (mTORC1) and mTOR complex 2 (mTORC2). Each of these complexes carries out distinct cellular functions and each complex is composed of several subunits. The most notable subunit of mTORC1 is the regulatory-associated protein of mTOR (Raptor) and in mTORC2, the rapamycin-insensitive companion of mTOR (Rictor) [52], [53], [54]. Most reports indicate that only mTORC1 is rapamycin sensitive, but some recent evidence suggests that, depending on the cell type, duration, and dosing regimen, rapamycin can also inhibit mTORC2 [55]. Downstream of mTORC1 is the ribosomal protein S6 kinases and its downstream substrate S6, which gets phosphorylated upon mTOR activation. In order to help understand why rapamycin did not suppress the allergic responses in our studies, we measured the activation of P-S6 downstream of mTORC1. In both of the models used here, rapamycin completely suppressed HDM-induced increases in phosphorylated S6 levels, but did not suppress the phosphorylation of Akt (S473). These results indicate that the dose of rapamycin used was sufficient to block mTORC1, but not mTORC2. These mTOR complexes differentially regulate T cell lineage commitment; with Th1 and Th17 being mostly dependent on mTORC1 signaling and Th2 cells on mTORC2 [11]. Accordingly, the Th1 cytokine IFN-γ and Th17 cytokine, IL-17A, were significantly decreased or trended lower in both of our models following rapamycin treatment whereas the prototypical Th2 cytokine IL-4 was increased. This increase in IL-4 is potentially the result of decreases in IFN-γ, a negative regulator of Th2 differentiation. Finally, IL-4 has been implicated in allergic responses independently of IL-13 [31], [56]. Taken together, regardless of the cellular source of IL-4 (Th2 cells, basophils, mast cells and/or eosinophils) increased pulmonary IL-4 levels may, at least partially, account for the lack of effect of rapamycin treatment on AHR and BALF eosinophilia.

In conclusion, while our earlier studies demonstrate that mTOR signaling plays an important role during the early phases of allergic asthma [18], the studies we report here suggest that its role is more limited during allergen re-exposure and chronic/established disease. This is consistent with studies showing a role for mTOR in early activation and differentiation events [11], [13], but it appears that once this is established, mTOR signaling plays a more minor role. It is possible that at these later stages of the disease process, other cells and mechanisms are driving the airway disease.

## Supporting Information

Figure S1 **HDM-specific IgG1 levels.** *A*, Protocol 1: Re-Exposure Study: HDM-specific IgG1 levels were increased in HDM rest and all groups re-exposed to HDM. Neither rapamycin (Rapa) nor dexamethasone (Dex) suppressed these increases (*n = *4–12 mice/group). *p<0.05 versus saline. *B*, Protocol 2: Reversal Study: HDM-specific IgG1 levels were increased after 3 and 6 weeks of HDM exposure. Both Rapa and Dex attenuated or suppressed this increase (*n = *3–8 mice/group). *p<0.05 versus saline; ∧*p*<0.05 versus vehicle; #*p*<0.05 versus dex.
(TIFF)

Figure S2 **Spleen weight to body weight ratios and lung cell populations.** Protocol 1 (Re-exposure): *A*, Spleen weights were increased after HDM re-exposure compared to saline controls. Rapamycin (Rapa) and dexamethasone (Dex) suppressed the increase in spleen weights (*n = *4–12 mice/group). *p<0.05 versus saline; ∧*p*<0.05 versus HDM. *B*, Total lung cells were increased after HDM re-exposure (group 3) compared to saline controls. Rapa and Dex suppressed total lung cells (*n = *4–12 mice/group). *p<0.05 versus saline; ∧*p*<0.05 versus HDM. *C*, HDM-induced increases in total CD3^+^ T cells after allergen re-exposure were suppressed by Rapa and Dex (*n = *4–12 mice/group). *p<0.05 versus saline; ∧*p*<0.05 versus HDM re-exposed. *D*, Total lung B cells after HDM re-exposure and after Rapa showed trends towards increased and decreased, respectively, but these changes did not reach statistical significance (*n = *4–12 mice/group). Protocol 2 (Chronic Allergen/Reversal): *E,* Spleen weights were increased after 6 weeks of HDM exposure and suppressed by Rapa and Dex (*n = *6–8 mice/group). *p<0.05 versus saline; ∧*p*<0.05 versus HDM; #*p*<0.05 versus Rapa. *F*, Total lung cells were increased after 6 weeks of HDM. Dex, but not Rapa suppressed this response (*n = *4–12 mice/group). *p<0.05 versus saline; ∧*p*<0.05 versus HDM; #*p*<0.05 versus Rapa. *G,* HDM-induced increases in CD3^+^ lung T cells after 6 weeks of HDM exposure were attenuated by Rapa and Dex (*n = *4–12 mice/group). *p<0.05 versus saline; ∧*p*<0.05 versus HDM re-exposed. *H*, Total lung B cells were increased after 6 weeks of HDM compared to saline controls, but were only significantly reduced after Dex, not Rapa (*n = *4–12 mice/group). *p<0.05 versus saline; ∧*p*<0.05 versus HDM re-exposed.
(TIFF)

Figure S3 **Protocol 2- HDM-specific IgE levels, inflammatory BALF cell numbers, and AHR after chronic HDM exposure.** *A*, Increases in HDM-specific IgE were observed after both 3 and 6 weeks of HDM. HDM-specific IgE levels were reduced by rapamycin (Rapa) and dexamethasone (Dex) treatment (*n = *5–9 mice/group). *p<0.05 versus saline; ∧*p*<0.05 versus HDM. *B*, Total BALF cell numbers were increased after 3 and 6 weeks of HDM exposure compared to saline controls and were even higher after Rapa treatment compared to HDM. Total BALF cell numbers were decreased in Dex treated mice compared to HDM mice (*n = *10–16 mice/group. *p<0.05 versus saline; ∧*p*<0.05 versus HDM; #*p*<0.05 versus Dex. *C*, Total macrophages and eosinophils were higher with Rapa treatment compared to mice exposed to HDM for 6 weeks. Neutrophil and eosinophil numbers were reduced in Dex treated mice compared to HDM (6 weeks) exposed mice (*n = *10–16 mice/group). *p<0.05 versus saline; ∧*p*<0.05 versus HDM; #*p*<0.05 versus Dex. *D*, The percentage of eosinophils was elevated in Rapa treated mice compared to saline controls, but similar to mice exposed to HDM for 6 weeks, whereas the percentage of eosinophils was decreased with Dex treatment (*n = *10–16 mice/group). *p<0.05 versus saline; ∧*p*<0.05 versus HDM; #*p*<0.05 versus Dex. *E*, AHR was increased after 3 weeks and 6 weeks of HDM exposure. Increases in AHR after 6 weeks of HDM exposure were not suppressed by Rapa or Dex at 50 mg/ml methacholine. However, at 25 mg/ml methacholine, Dex did reduce AHR compared to mice exposed to HDM for 6 weeks (*n = *10–16 mice/group). *p<0.05 versus saline.
(TIFF)

Figure S4 **Protocol 2- Goblet cell markers in the lungs after chronic HDM exposure.** *A*, CLCA3 protein in lung homogenates was increased after 3 and 6 weeks of HDM exposure, was unaltered by rapamycin (Rapa), but was suppressed by dexamethasone (Dex) (*n = *4–8 mice/group). *p<0.05 versus saline; ∧*p*<0.05 versus HDM. *B*, SPDEF levels were also increased after 6 weeks of HDM exposure, but unaltered by Rapa. SPDEF levels were lower with Dex treatment compared to HDM exposed mice, but this did not reach statistical significance (*n = *3 mice/group). *p<0.05 versus saline. *C*, Muc5AC staining was increased in the airway epithelial cells after 3 and 6 weeks of HDM exposure. Dex attenuated these increases, but Rapa did not.
(TIFF)

Figure S5 **Airway smooth muscle staining in allergic asthma models.** *A*, Protocol 1 (Re-exposure): α-Smooth muscle actin (α-SMA) staining was performed on lung sections of mice re-exposed to HDM after 6 weeks of rest. Similar staining patterns were observed between all animal groups with no observable differences between rapamycin (Rapa) and dexamethasone (Dex) treated mice. *B*, Protocol 2 (Chronic Allergen/Reversal): α-Smooth muscle actin (α-SMA) staining in the lung after 6 weeks of saline or HDM exposure was similar. No differences were observed with Rapa or Dex treatment.
(TIFF)

Figure S6 **Protocol 2- Western blot analysis of P-S6 and P-Akt levels after chronic HDM exposure.** *A*, P-S6, a downstream target of mTOR complex 1, was increased after both 3 and 6 weeks of HDM exposure. Rapamycin (Rapa) treatment during weeks 4–6 of HDM exposure completely suppressed this increase. Levels of P-S6 were also reduced in the lung by dexamethasone (Dex) (*n = *3–5 mice/group). *p<0.05 versus saline; ∧*p*<0.05 versus HDM; #*p*<0.05 versus Dex. *B*, Levels of P-Akt, a downstream target of mTOR complex 2, were not significantly altered by 6 weeks of HDM alone, Rapa, or Dex treatment (*n = *3–5 mice/group).
(TIFF)

Figure S7 **Activated CD69^+^Foxp3** ^−^ **T cells in the lungs of mice.** *A*, Protocol 1 (Re-exposure): FACS analysis showing increases in CD69^+^Foxp3^−^ T cells after HDM re-exposure. CD69^+^Foxp3^−^ T cells were reduced with rapamycin (Rapa) and dexamethasone (Dex) treatment. *B*, Protocol 2 (Chronic Allergen/Reveral): FACS analysis demonstrating increases in CD69^+^Foxp3^−^ T cells after chronic HDM exposure. Slight reductions were observed after Rapa and Dex treatment.
(TIFF)
