# Supplementary figures and images for: Differential Effects of Rapamycin and Dexamethasone in Mouse Models of Established Allergic Asthma
Source: PLoS One. 2013 Jan 17;8(1):e54426. doi: 10.1371/journal.pone.0054426 (PMC3547928; doi:10.1371/journal.pone.0054426)

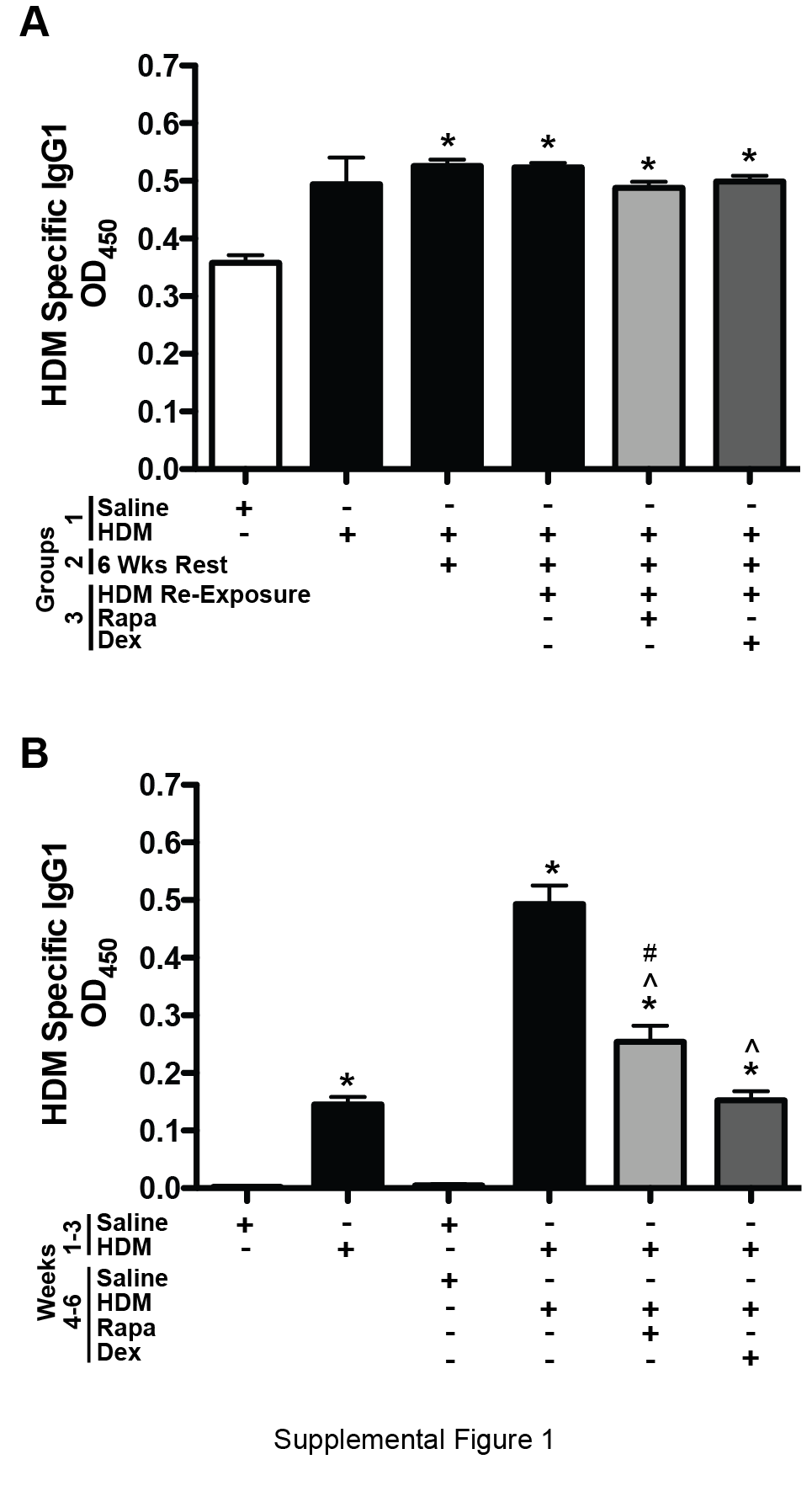

Supplement: Figure S1 — HDM-specific IgG1 levels. A, Protocol 1: Re-Exposure Study: HDM-specific IgG1 levels were increased in HDM rest and all groups re-exposed to HDM. Neither rapamycin (Rapa) nor dexamethasone (Dex) suppressed these increases (n = 4–12 mice/group). *p<0.05 versus saline. B, Protocol 2: Reversal Study: HDM-specific IgG1 levels were increased after 3 and 6 weeks of HDM exposure. Both Rapa and Dex attenuated or suppressed this increase (n = 3–8 mice/group). *p<0.05 versus saline; ∧p<0.05 versus vehicle; #p<0.05 versus dex. (TIFF) [file pone.0054426.s001.tiff]

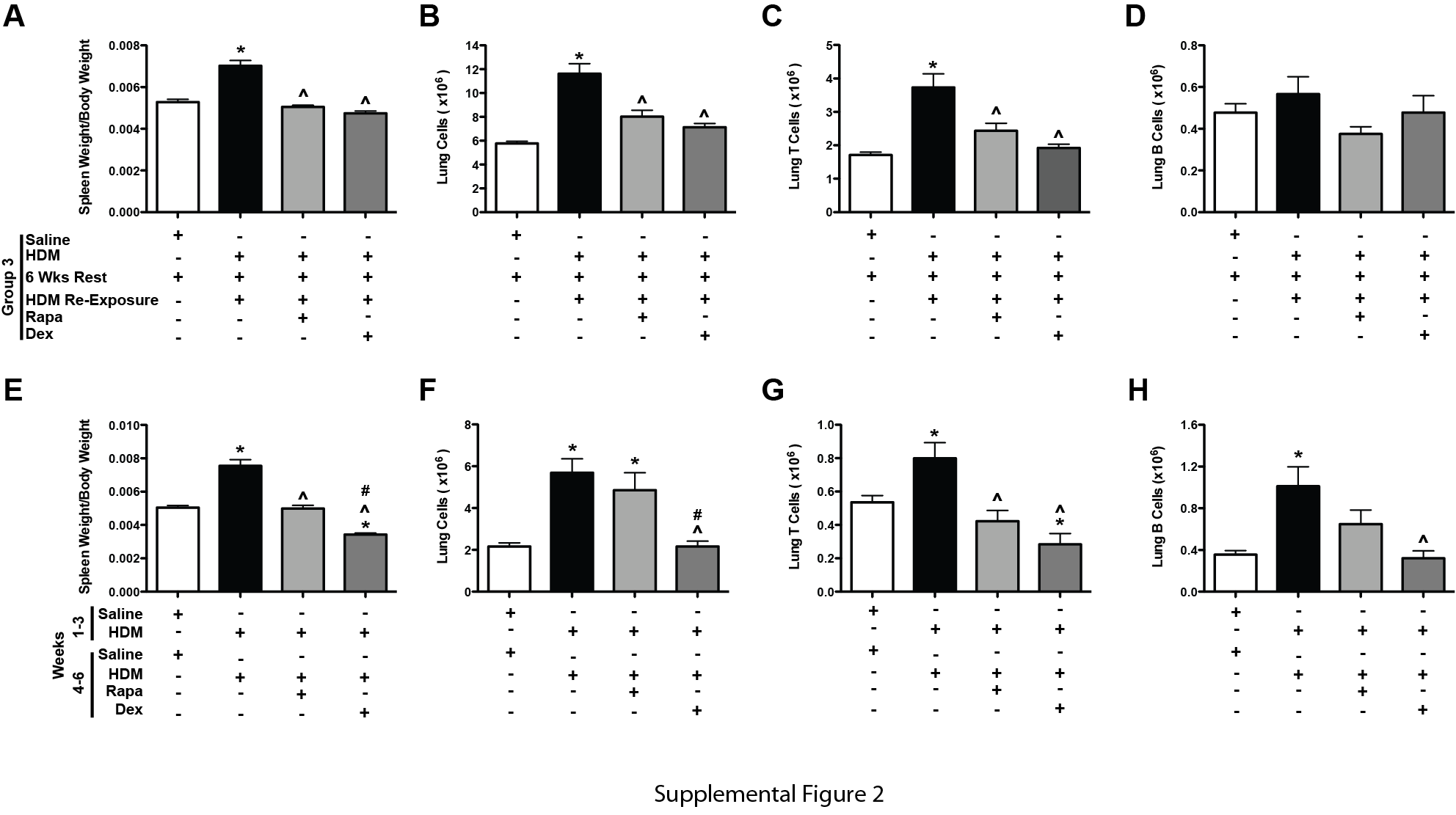

Supplement: Figure S2 — Spleen weight to body weight ratios and lung cell populations. Protocol 1 (Re-exposure): A, Spleen weights were increased after HDM re-exposure compared to saline controls. Rapamycin (Rapa) and dexamethasone (Dex) suppressed the increase in spleen weights (n = 4–12 mice/group). *p<0.05 versus saline; ∧p<0.05 versus HDM. B, Total lung cells were increased after HDM re-exposure (group 3) compared to saline controls. Rapa and Dex suppressed total lung cells (n = 4–12 mice/group). *p<0.05 versus saline; ∧p<0.05 versus HDM. C, HDM-induced increases in total CD3+ T cells after allergen re-exposure were suppressed by Rapa and Dex (n = 4–12 mice/group). *p<0.05 versus saline; ∧p<0.05 versus HDM re-exposed. D, Total lung B cells after HDM re-exposure and after Rapa showed trends towards increased and decreased, respectively, but these changes did not reach statistical significance (n = 4–12 mice/group). Protocol 2 (Chronic Allergen/Reversal): E, Spleen weights were increased after 6 weeks of HDM exposure and suppressed by Rapa and Dex (n = 6–8 mice/group). *p<0.05 versus saline; ∧p<0.05 versus HDM; #p<0.05 versus Rapa. F, Total lung cells were increased after 6 weeks of HDM. Dex, but not Rapa suppressed this response (n = 4–12 mice/group). *p<0.05 versus saline; ∧p<0.05 versus HDM; #p<0.05 versus Rapa. G, HDM-induced increases in CD3+ lung T cells after 6 weeks of HDM exposure were attenuated by Rapa and Dex (n = 4–12 mice/group). *p<0.05 versus saline; ∧p<0.05 versus HDM re-exposed. H, Total lung B cells were increased after 6 weeks of HDM compared to saline controls, but were only significantly reduced after Dex, not Rapa (n = 4–12 mice/group). *p<0.05 versus saline; ∧p<0.05 versus HDM re-exposed. (TIFF) [file pone.0054426.s002.tiff]

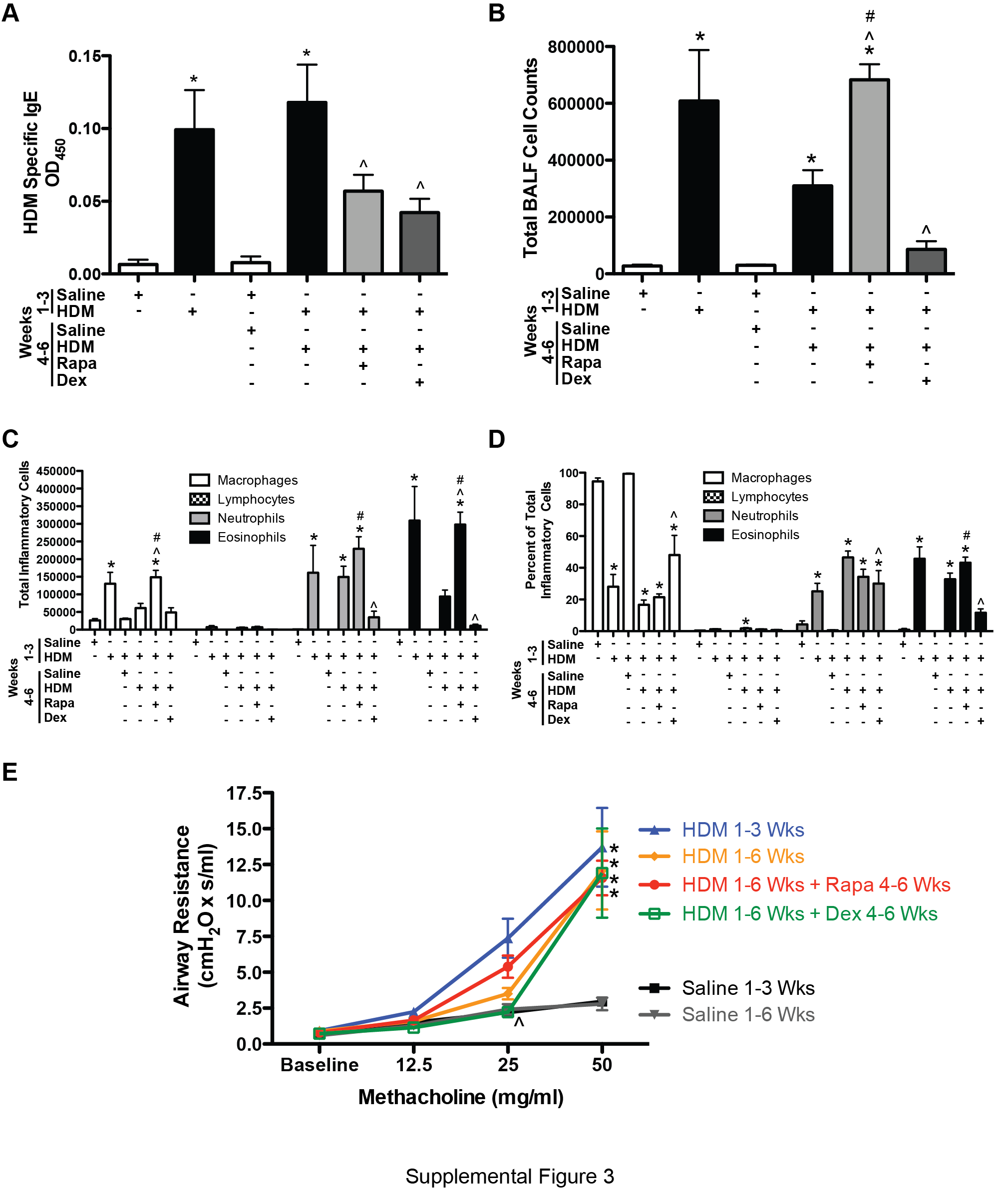

Supplement: Figure S3 — Protocol 2- HDM-specific IgE levels, inflammatory BALF cell numbers, and AHR after chronic HDM exposure. A, Increases in HDM-specific IgE were observed after both 3 and 6 weeks of HDM. HDM-specific IgE levels were reduced by rapamycin (Rapa) and dexamethasone (Dex) treatment (n = 5–9 mice/group). *p<0.05 versus saline; ∧p<0.05 versus HDM. B, Total BALF cell numbers were increased after 3 and 6 weeks of HDM exposure compared to saline controls and were even higher after Rapa treatment compared to HDM. Total BALF cell numbers were decreased in Dex treated mice compared to HDM mice (n = 10–16 mice/group. *p<0.05 versus saline; ∧p<0.05 versus HDM; #p<0.05 versus Dex. C, Total macrophages and eosinophils were higher with Rapa treatment compared to mice exposed to HDM for 6 weeks. Neutrophil and eosinophil numbers were reduced in Dex treated mice compared to HDM (6 weeks) exposed mice (n = 10–16 mice/group). *p<0.05 versus saline; ∧p<0.05 versus HDM; #p<0.05 versus Dex. D, The percentage of eosinophils was elevated in Rapa treated mice compared to saline controls, but similar to mice exposed to HDM for 6 weeks, whereas the percentage of eosinophils was decreased with Dex treatment (n = 10–16 mice/group). *p<0.05 versus saline; ∧p<0.05 versus HDM; #p<0.05 versus Dex. E, AHR was increased after 3 weeks and 6 weeks of HDM exposure. Increases in AHR after 6 weeks of HDM exposure were not suppressed by Rapa or Dex at 50 mg/ml methacholine. However, at 25 mg/ml methacholine, Dex did reduce AHR compared to mice exposed to HDM for 6 weeks (n = 10–16 mice/group). *p<0.05 versus saline. (TIFF) [file pone.0054426.s003.tiff]

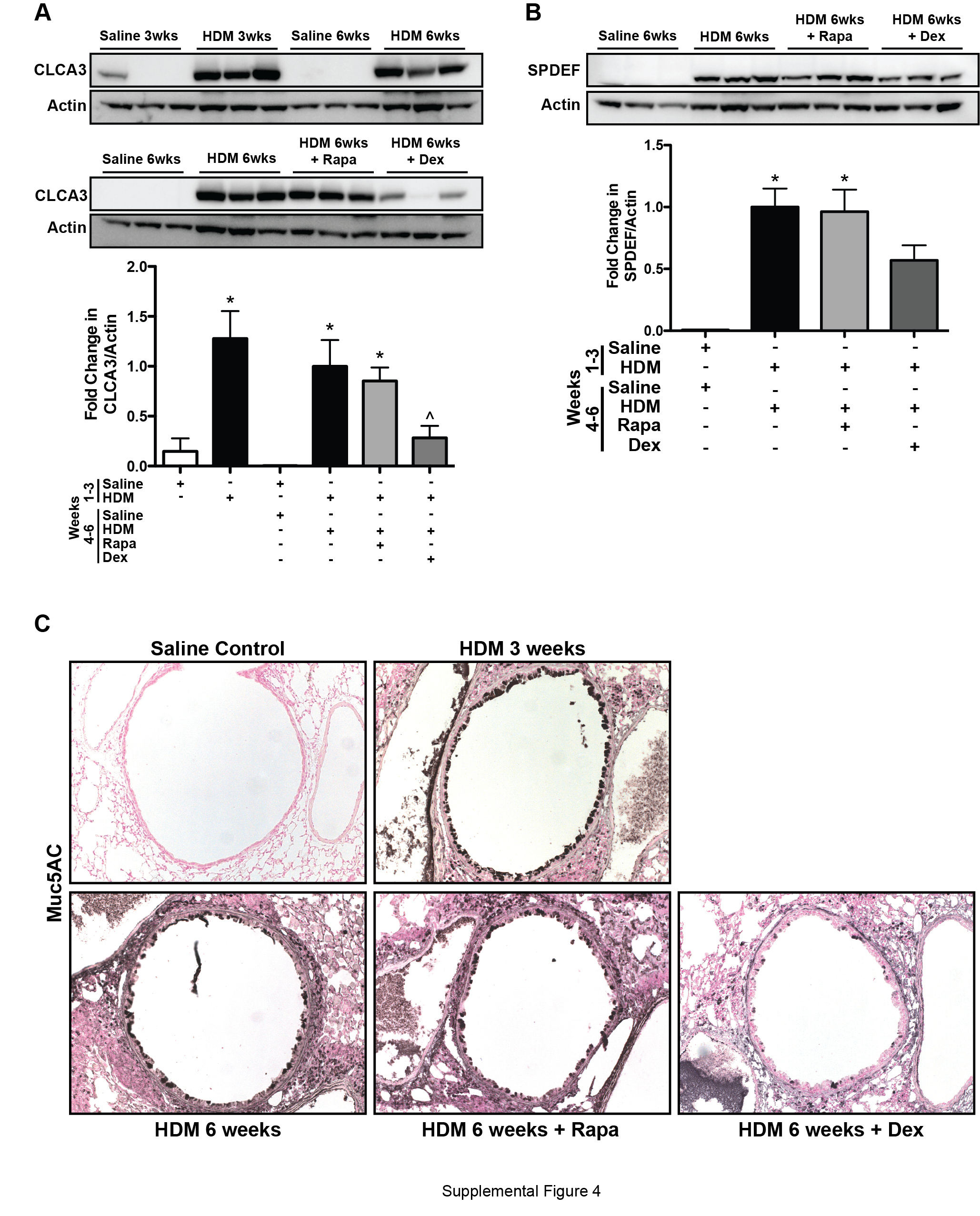

Supplement: Figure S4 — Protocol 2- Goblet cell markers in the lungs after chronic HDM exposure. A, CLCA3 protein in lung homogenates was increased after 3 and 6 weeks of HDM exposure, was unaltered by rapamycin (Rapa), but was suppressed by dexamethasone (Dex) (n = 4–8 mice/group). *p<0.05 versus saline; ∧p<0.05 versus HDM. B, SPDEF levels were also increased after 6 weeks of HDM exposure, but unaltered by Rapa. SPDEF levels were lower with Dex treatment compared to HDM exposed mice, but this did not reach statistical significance (n = 3 mice/group). *p<0.05 versus saline. C, Muc5AC staining was increased in the airway epithelial cells after 3 and 6 weeks of HDM exposure. Dex attenuated these increases, but Rapa did not. (TIFF) [file pone.0054426.s004.tiff]

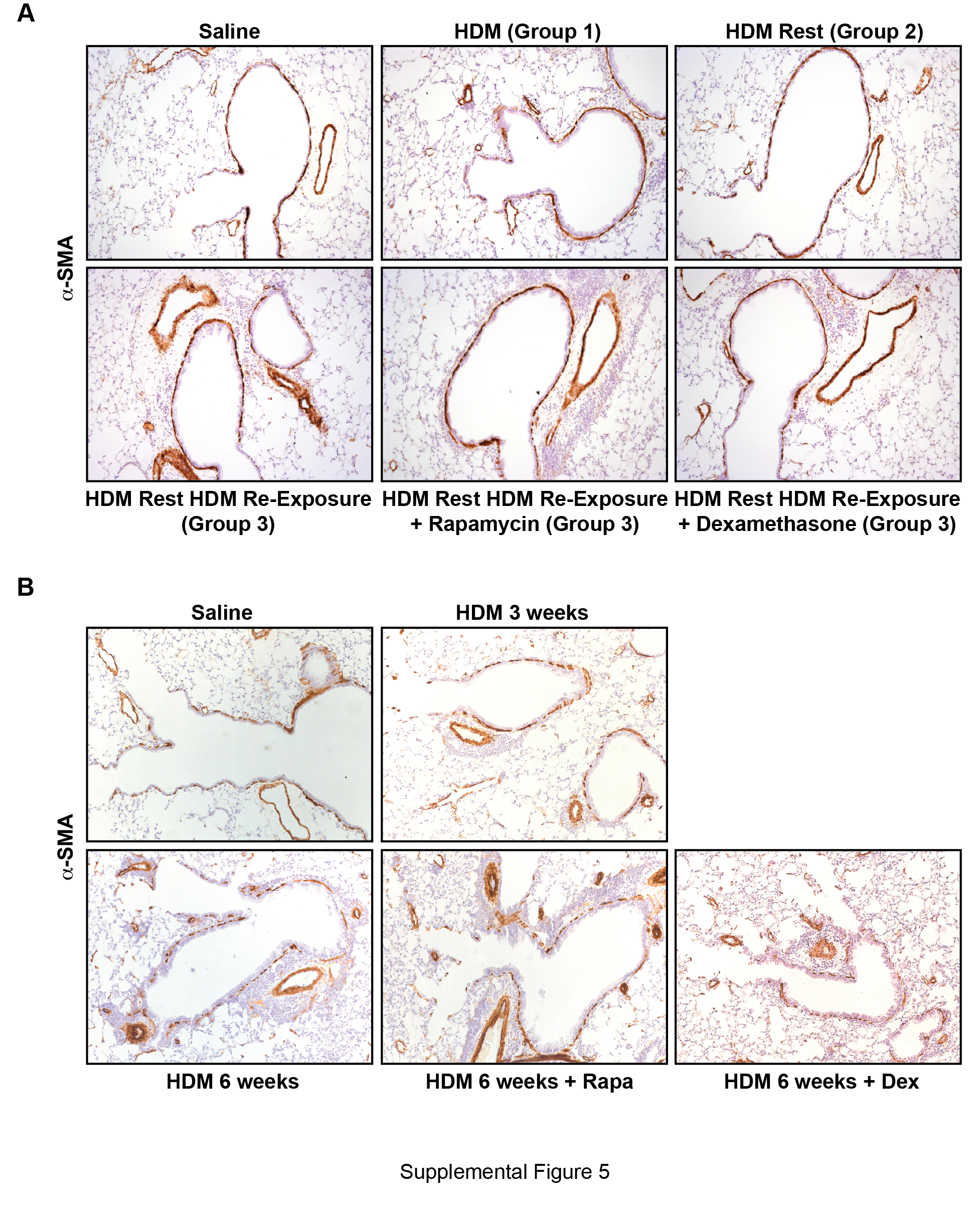

Supplement: Figure S5 — Airway smooth muscle staining in allergic asthma models. A, Protocol 1 (Re-exposure): α-Smooth muscle actin (α-SMA) staining was performed on lung sections of mice re-exposed to HDM after 6 weeks of rest. Similar staining patterns were observed between all animal groups with no observable differences between rapamycin (Rapa) and dexamethasone (Dex) treated mice. B, Protocol 2 (Chronic Allergen/Reversal): α-Smooth muscle actin (α-SMA) staining in the lung after 6 weeks of saline or HDM exposure was similar. No differences were observed with Rapa or Dex treatment. (TIFF) [file pone.0054426.s005.tiff]

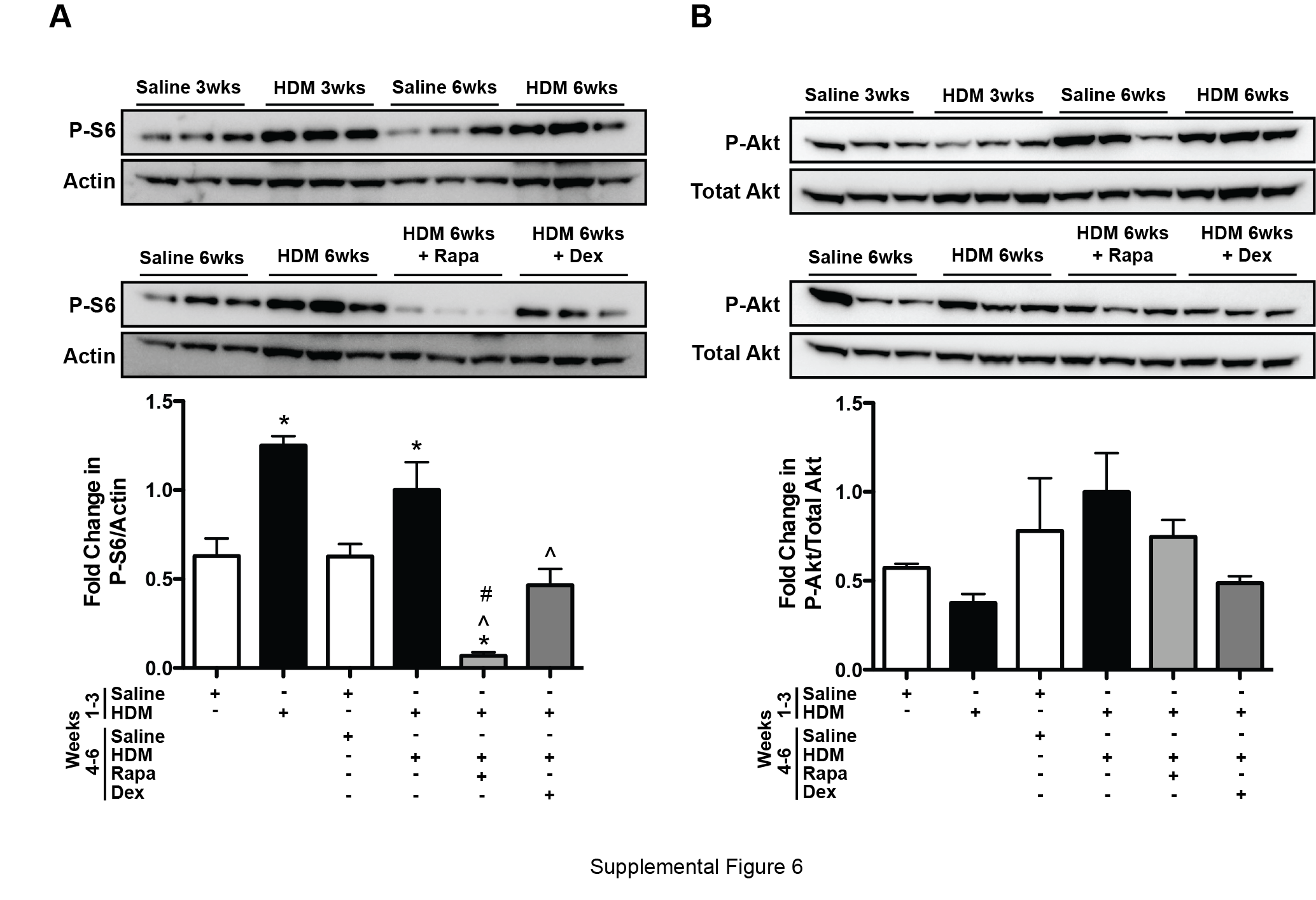

Supplement: Figure S6 — Protocol 2- Western blot analysis of P-S6 and P-Akt levels after chronic HDM exposure. A, P-S6, a downstream target of mTOR complex 1, was increased after both 3 and 6 weeks of HDM exposure. Rapamycin (Rapa) treatment during weeks 4–6 of HDM exposure completely suppressed this increase. Levels of P-S6 were also reduced in the lung by dexamethasone (Dex) (n = 3–5 mice/group). *p<0.05 versus saline; ∧p<0.05 versus HDM; #p<0.05 versus Dex. B, Levels of P-Akt, a downstream target of mTOR complex 2, were not significantly altered by 6 weeks of HDM alone, Rapa, or Dex treatment (n = 3–5 mice/group). (TIFF) [file pone.0054426.s006.tiff]

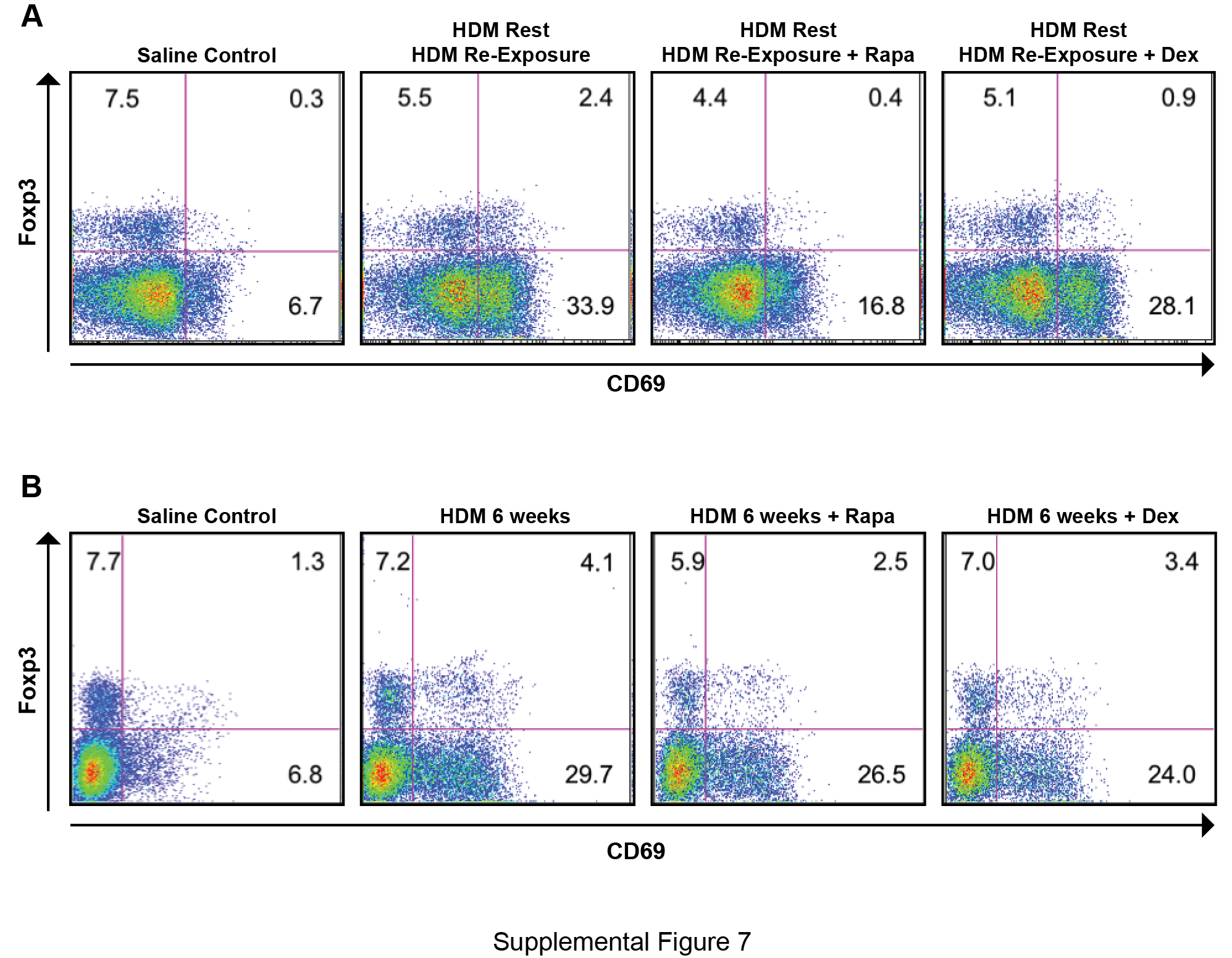

Supplement: Figure S7 — Activated CD69+Foxp3 − T cells in the lungs of mice. A, Protocol 1 (Re-exposure): FACS analysis showing increases in CD69+Foxp3− T cells after HDM re-exposure. CD69+Foxp3− T cells were reduced with rapamycin (Rapa) and dexamethasone (Dex) treatment. B, Protocol 2 (Chronic Allergen/Reveral): FACS analysis demonstrating increases in CD69+Foxp3− T cells after chronic HDM exposure. Slight reductions were observed after Rapa and Dex treatment. (TIFF) [file pone.0054426.s007.tiff]
